# Intramolecular Ring‐Expansion Reaction (RER) and Intermolecular Coordination of In Situ Generated Cyclic (Amino)(aryl)carbenes (cAArCs)

**DOI:** 10.1002/chem.201902630

**Published:** 2019-08-02

**Authors:** Jan Lorkowski, Mirjam Krahfuß, Maciej Kubicki, Udo Radius, Cezary Pietraszuk

**Affiliations:** ^1^ Faculty of Chemistry Department of Organometallic Chemistry Adam Mickiewicz University in Poznań ul. Uniwersytetu Poznańskiego 8 61-614 Poznań Poland; ^2^ Institut für Anorganische Chemie Julius-Maximilians-Universität Würzburg Am Hubland 97074 Würzburg Germany

**Keywords:** cAArC, complexes, copper, NHC, ring-expansion reaction

## Abstract

Cyclic (amino)(aryl)carbenes (cAArCs) based on the isoindoline core were successfully generated in situ by α‐elimination of 3‐alkoxyisoindolines at high temperatures or by deprotonation of isoindol‐2‐ium chlorides with sodium or copper(I) acetates at low temperatures. 3‐Alkoxy‐isoindolines **2 a**,**b‐OR** (R=Me, Et, *i*Pr) have been prepared in high yields by the addition of a solution of 2‐aryl‐1,1‐diphenylisoindol‐2‐ium triflate (**1 a**,**b‐OTf**; **a**: aryl=Dipp=2,6‐diisopropylphenyl; **b**: Mesityl‐, Mes=2,4,6‐trimethylphenyl) to the corresponding alcohol (ROH) with NEt_3_ at room temperature. Furthermore, the reaction of **2 a**,**b‐OMe** in diethyl ether with a tenfold excess of hydrochloric acid led to the isolation of the isoindol‐2‐ium chlorides **1 a**,**b‐Cl** in high yields. The thermally generated cAArC reacts with sulfur to form the thioamide **3 a**. Without any additional trapping reagent, *in situ* generation of 1,1‐diphenylisoidolin‐3‐ylidenes does not lead to the isolation of these compounds, but to the reaction products of the insertion of the carbene carbon atom into an *ortho* C−H bond of a phenyl substituent, followed by ring‐expansion reaction; namely, anthracene derivatives 9‐N(H)aryl‐10‐Ph‐C_14_H_8_ **4 a**,**b** (**a**: Dipp; **b**: Mes). These compounds are conveniently synthesized by deprotonation of the isoindol‐2‐ium chlorides with sodium acetate in high yields. Deprotonation of **1 a‐Cl** with copper(I) acetate at low temperatures afforded a mixture of **4 a** and the corresponding cAArC copper(I) chloride **5 a**, and allowed the isolation and structural characterization of the first example of a cAArC copper complex of general formula [(cAArC)CuCl].

## Introduction

**Scheme 1:**
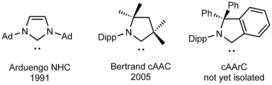
Examples of an N‐heterocyclic carbene (NHC), a cyclic (alkyl)(amino)carbene (cAAC) and a cyclic (amino)(aryl)carbene (cAArC) (Ad=adamantyl; Dipp=2,6‐diisopropylphenyl).

After the discovery of the first stable (phosphino)(silyl)carbene in 1988^1^ by Bertrand et al. and the subsequent successful synthesis and isolation of the first stable crystalline N‐heterocyclic carbene (NHC), 1,3‐diadamantyl‐imidazolin‐2‐ylidene, in 1991 by Arduengo et al.^2^ carbenes have been heavily applied in main‐group element^3^ and transition‐metal chemistry,^4^ as well as in organocatalysis.^5^ The efficiency of NHCs and related molecules in these fields is mostly attributed to the sterically demanding structure and strong σ‐donor properties of these compounds.^6^ Notably, these parameters can be easily adjusted by changing the substitution pattern at the nitrogen and carbene carbon atoms, and thus there is an increasing interest towards new carbenes with “superior” stereoelectronic properties. For example, Bertrand et al. reported in 2005 on the synthesis of another class of carbenes called cyclic (alkyl)(amino)carbenes (cAACs), which lately has attracted an enormous attention.^7^ The structure of cAACs (see Scheme 1) originates from the replacement of one of the electronegative amine substituents of NHCs by a stronger σ‐donor methylene group, which renders them as one of the most nucleophilic and simultaneously the most electrophilic isolable five‐membered ring carbenes. Isoindoline‐3‐ylidenes are close derivatives of cAACs, which result from the formal replacement of the alkyl substituents of cAACs by an aryl group (Scheme 1).

**Scheme 2:**
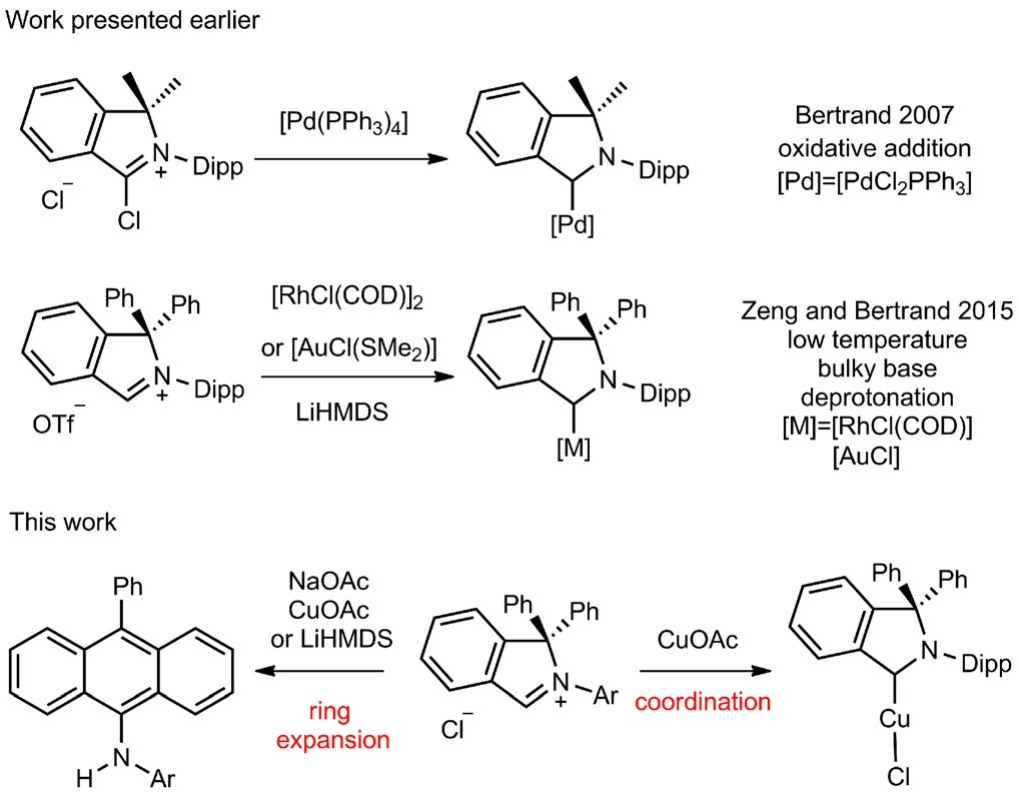
Methods of cAArC metal complexes preparation.

This class of carbenes, called cyclic (amino)(aryl)carbenes (cAArCs) was introduced by Bertrand over a decade ago.^8^ It was reported that C‐chloroiminium salts based on the isoindoline core, precursor of these cAArCs, can undergo oxidative addition to a palladium(0) center, which resulted in the formation of an isolable cAArC palladium complex. The generation of an unstable, uncoordinated cAArC was avoided in this process, a problem that was only recently revised by the same group. In 2015 Zeng and Bertrand et al. successfully generated a series of isoindoline‐3‐ylidenes by deprotonation of isoindol‐2‐ium triflates at low temperatures and trapped these molecules in the coordination sphere of rhodium and gold, forming air‐stable complexes (Scheme 2).^9^ Furthermore, it has been demonstrated that the electronic properties of isoindoline‐3‐ylidenes are characterized by an enhanced electrophilicity of the carbene carbon atom relative to cAACs, whereas the high nucleophilicity is retained due to the narrow HOMO–LUMO energy gap.

Notably, the low singlet–triplet transition energy is one of the obstacles that prevents the yet not achieved isolation of free cAArCs, limiting the application of these interesting singlet carbenes. Another roadblock for the cAACs utilization, as investigations on cAArC generation by deprotonation of different iminium salts revealed,^10^ lies in the high electrophilicity due to an energetically low‐lying carbon‐centered acceptor orbital of these carbene precursors. This feature results in a higher susceptibility towards nucleophilic attack compared to rapid deprotonation, and thus even the application of many bases generally considered to be “non‐nucleophilic” led to the formation of stable iminium‐base adducts, rather than to C−H deprotonation of the iminium salt.^11^ For example, attempts of cAArC generation by deprotonation of the phenanthridinium salt with sodium *tert*‐butoxide, lithium hexamethyldisilazane and even mesityl lithium were ineffective and resulted exclusively in the nucleophilic addition of the base to the iminium salt.^11^ However, these “base adducts” might also be suitable for carbene synthesis by an intramolecular deprotonation route, as has been demonstrated for the generation of isolable NHCs.^12^ For example, the preparation of Wanzlick dimers has been reported from an appropriate chloroform NHC adduct,12a and the first “bottleable” triazol‐5‐ylidene was prepared by Enders et al. by α‐elimination of methanol from the corresponding methoxy ether.12b On the other hand, carbene complex formation by deprotonation may also be achieved using a metal salt of simple basic counter‐anions, in which the metal salt serves as both metal source and deprotonation reagent.^13^ In fact, the first synthesis of an NHC metal complex reported by Wanzlick et al. has been achieved using mercury acetate and the corresponding iminium salt as the carbene precursor with the elimination of acetic acid.^14^ Since then, this methodology has been applied for the preparation of many NHC metal complexes,^13^ and some effort has been made in recent years to apply this method in combination with subsequent NHC transfer to avoid the generation of free carbenes (and the inert atmosphere required therefore).^15^ Iminium salt deprotonation with simple basic copper salts such as copper(I) oxide or copper(I) acetate at elevated temperatures allows the straightforward preparation of a plethora of these valuable compounds, and this approach was also found applicable for highly reactive carbenes like CAACs, abnormal NHCs (aNHCs) and mesoionic carbenes (MICs).^16^

Herein, our recent investigations aiming at the generation of free isoindoline‐3‐ylidenes (cAArCs) starting from 3‐alkoxy‐isoindolines, as well as iminium chlorides in the presence of metal salts with basic anions are presented (Scheme 2). The use of these disruptive methods led to the isolation of both, unique intramolecular ring‐expansion reaction products **4 a**,**b** and the first copper complex of cyclic (amino)(aryl)carbenes (**5 a**).

## Results and Discussion

**Scheme 3:**
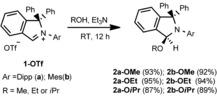
Synthesis of 2‐aryl‐3‐alkoxy‐1,1‐diphenylisoindolines **2 a** and **2 b**.

**Figure 1:**
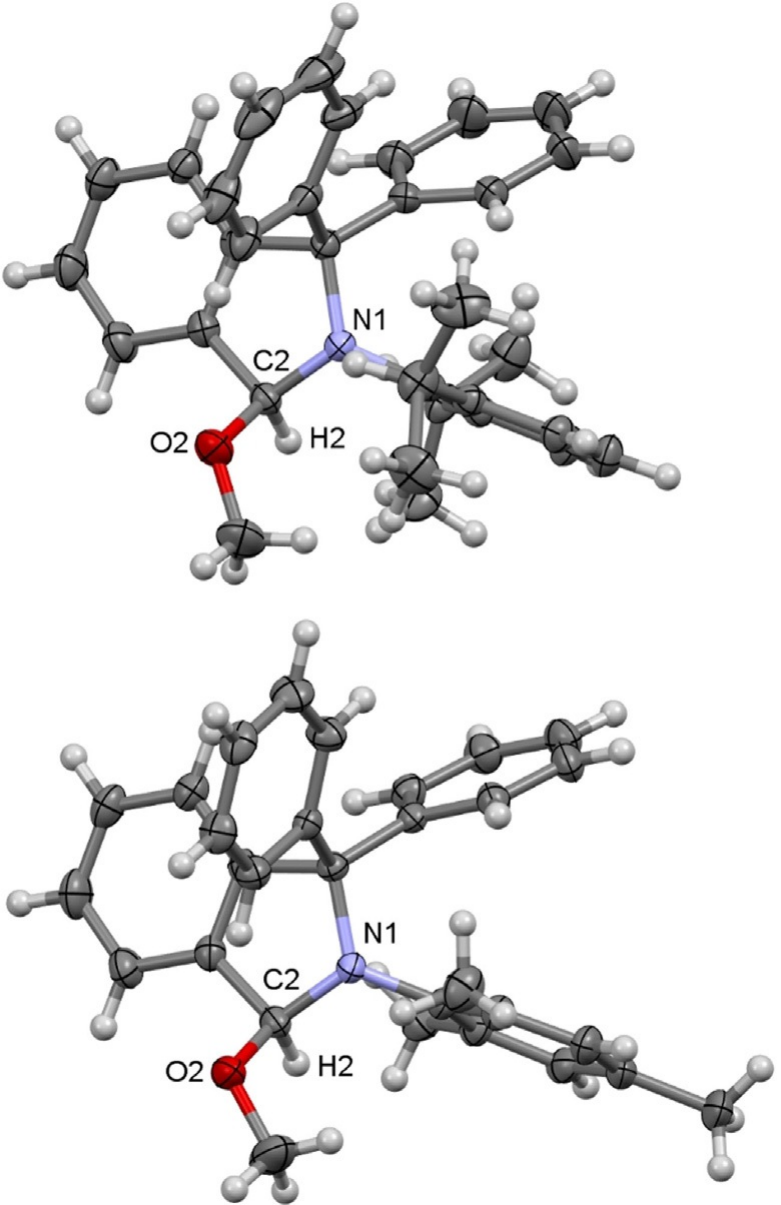
Molecular structure of **2 a‐OMe** (top) and **2 b‐OMe** (bottom**)** in the solid state (ellipsoids set at the 33 % probability level). Selected bond lengths [Å] and angles [°] of **2 a‐OMe**: N1−C2 1.443 (2), C2−H2 0.98, C2−O2 1.437 (2); H2‐C2‐O2 101, Selected bond lengths [Å] and angles [°] of **2 b‐OMe**: N1−C2 1.444 (3), C2−H2 0.98, C2−O2 1.429 (3); H2‐C2‐O2 110.

**Figure 2:**
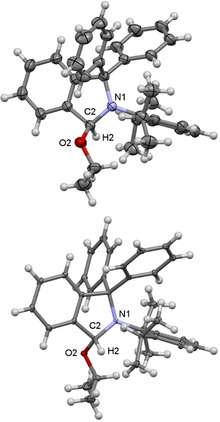
Molecular structure of **2 a‐OEt** (top) and **2 a‐O*i*Pr** (bottom) in the solid state (ellipsoids set at the 33 % probability level). Selected bond lengths [Å] and angles [°] of **2 a‐OEt**: N1−C2 1.446 (4), C2−H2 0.98, C2−O2 1.433 (4); H2‐C2‐O2 110. Selected bond lengths [Å] and angles [°] of **2 a‐O*i*Pr**: N1−C2 1.4481 (18), C2−H2 1.00, C2−O2 1.4301 (18); H2‐C2‐O2 110.

The starting point of our investigation was the synthesis of a series of simple 3‐alkoxy‐substituted isoindolines to evaluate their potential as cAArC precursors at elevated temperatures. 3‐Methoxy‐isoindolines (**2 a‐OMe** and **2 b‐OMe**) were prepared by treatment of a methanol solution of 2‐aryl‐1,1‐diphenylisoindol‐2‐ium triflates (**1 a**,**b‐OTf**; **a**: aryl=Dipp=2,6‐diisopropylphenyl; **b**: Mesityl‐, Mes=2,4,6‐trimethylphenyl) with NEt_3_ at room temperature, which led to the solvent deprotonation and formation of the hemiaminal ethers **2 a**,**b‐OMe** in 93 % and 92 % isolated yield, respectively (Scheme 3). The highest yields were obtained using anhydrous conditions and an excess of NEt_3_ (1.5 equiv) as a non‐nucleophilic base. Single‐crystals suitable for X‐ray diffraction have been crystallized from dichloromethane/methanol solutions of **2 a**,**b‐OMe** and the molecular structures of these derivatives confirm the attack of the methoxide at the iminium carbon atom (Figure 1). Similarly, the reactions of **1 a**,**b‐OTf** with NEt_3_ in ethanol and isopropanol with an excess of NEt_3_ resulted in the formation of the 3‐ethoxy and 3‐isopropoxy derivatives **2 a**,**b‐OEt** and **2 a**,**b‐O*i*Pr** in high yields. All compounds have been characterized using NMR spectroscopy and mass spectrometry, the results of X‐ray diffraction performed on single‐crystals of **2 a‐OEt** and **2 a‐O*i*Pr** are shown in Figure 2. The solid‐state structures of **2 a‐OEt** and **2 a‐O*i*Pr** confirm the connectivity of these molecules, the metric parameters of these compounds are not unexceptional.

**Figure 3:**
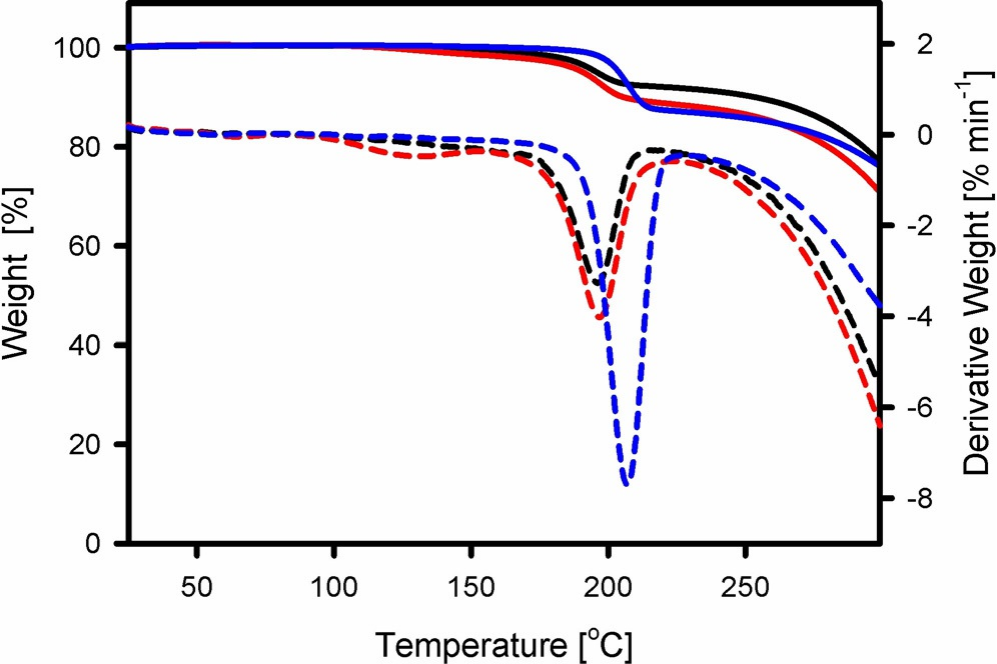
Thermogravimetric analysis of **2 a‐OMe** (black), **2 a‐OEt** (red) and **2 a‐O*i*Pr** (blue).

The series of compounds **2 a‐OR** (R=Me, Et, *i*Pr) were then tested for their use as precursors of isoindoline‐3‐ylidenes in thermal activation reactions, which should proceed by the α‐elimination of an alcohol molecule to give the free cAArC.12b The thermogravimetric analysis of samples of **2 a‐OR** reveals a characteristic mass loss indicating the elimination of the corresponding alcohol at temperatures above 190 °C (Figure 3). This result indicates the possibility of cAArCs generation through high temperature α‐elimination of an alcohol molecule starting from the 3‐alkoxy‐substituted isoindolines in the solid state. However, all attempts to synthesize the cAArCs from the corresponding hemiaminal ethers using this type of solid‐state reaction (heating hemiaminal ethers **2 a**,**b‐OR** under dynamic vacuum) led to the formation of the rearrangement product **4** (see below) and/or to decomposition, depending on the temperature used.

**Scheme 4:**
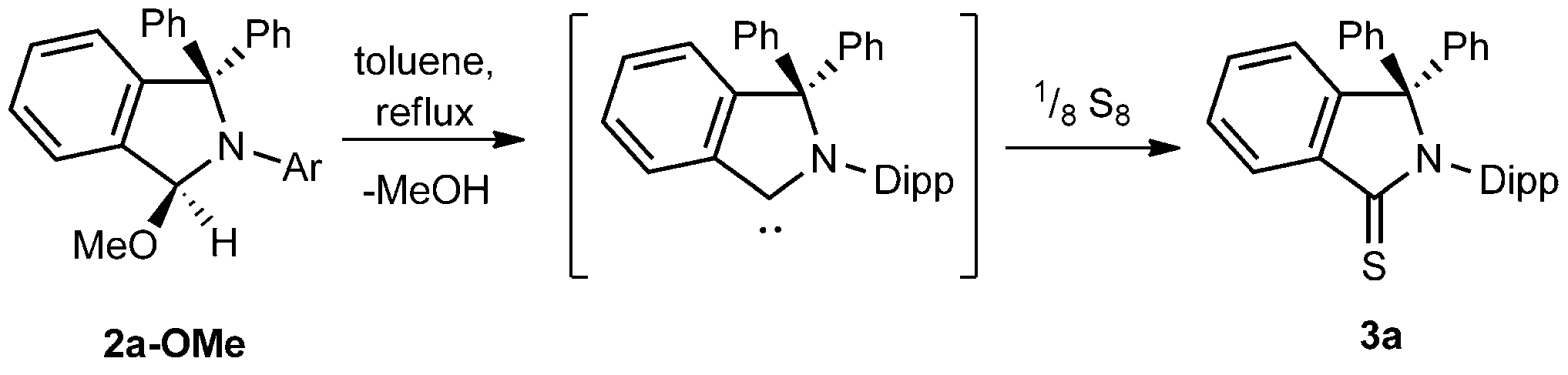
Formation of a thioamide **3 a**.

Compound **2 a‐OMe** was refluxed in toluene in the presence of elemental sulfur to further evaluate the concept of the high‐temperature isoindoline‐3‐ylidenes generation in solution. This reaction resulted in the high‐yield formation of the thioamide **3 a**, which was isolated in 92 % yield (Scheme 4). Sulphur serves here as scavenger of the free cAArC once formed, as has been demonstrated earlier for other carbenes, for example by the work of Wanzlick and Enders et al.^17^ Thus, a reasonable mechanistic interpretation of this reaction would be that the thermal treatment of the 3‐alkoxy‐substituted isoindolines **2 a**,**b‐OR** lead to the formation of traces of the cAArC, which then react with elemental sulfur (Scheme 4). Note that compound **3 a** has been synthesized previously by Bertrand et al. by deprotonation of **1 a‐OTf** with lithium bis(trimethylsilyl)amide in the presence of sulfur.^9^

Unfortunately, so far, we haven't found conditions to introduce cAArCs generated by α‐elimination of alcohols from 3‐alkoxy‐substituted isoindolines into the coordination sphere of transition metals. All reactions of **2 a**,**b‐OR** with various metal precursors, including the typical copper(I) precursors,^13^, ^14^, ^15^, ^16^ led to mixtures of products, which we were not able to separate. To establish a reason behind such a behavior, we attempted the preparation of free cAArCs by thermolysis of **2 a**,**b‐OR**. Overnight heating of toluene solutions of **2 a‐OMe** at 130 °C resulted in the formation of a yellow mixture consisting of starting material and a single new product in a 1 to 1 ratio, according to ^1^H NMR spectroscopy (Figure S29). The latter was the rearrangement product **4 a** (see below), which was isolated together with a considerable amount of starting material. However, assuming that a full conversion was not achieved due to an equilibrium between HOR α‐elimination from the 3‐alkoxy‐substituted isoindolines **2 a**,**b‐OR** and HOR addition to the carbene cAArC,^18^ we considered a further change in the reactions conditions.

**Scheme 5:**
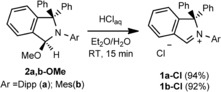
Synthesis of the isoindol‐2‐ium chlorides **1 a**,**b‐Cl**.

As has been shown before, the counterion exchange in imidazolium salts with formation of the corresponding chloride facilitates the heterolytic C−H bond cleavage due to chloride‐iminium proton hydrogen bonding, which makes these molecules more acidic.^19^ To extend this strategy to isoindol‐2‐ium salts, we developed a convenient protocol for a high‐yield counterion exchange starting from the readily available 3‐methoxy‐substituted isoindolines. The reaction of **2 a**,**b‐OMe** in diethyl ether with a tenfold excess of hydrochloric acid led to the isolation of the isoindol‐2‐ium chlorides **1 a**,**b‐Cl** in high yields (Scheme 5).

**Figure 4:**
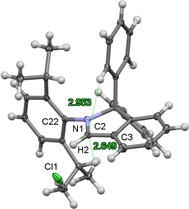
Molecular structure of **1 a‐Cl** in the solid state (ellipsoids set at the 33 % probability level). The distances between carbenic carbon atom and the protons prone to insertion are included (green dotted line [Å]). Selected bond lengths [Å] and angles [°]: N1−C2 1.297 (5), C2−H2 0.95, C2−C3 1.429 (6); N1‐C2‐H2 124, H2‐C2‐C3 124.

Remarkably, the synthesis of **1 a‐Cl** could be easily carried out starting directly from **1 a‐OTf** on a gram scale without the need for anhydrous conditions and the isolation of **2 a‐OMe**. It has previously been reported that this type of ion exchange in NHC salts requires application of ion exchange resins.^16^ In comparison, the method developed by us allows the convenient and quantitative conversion of **1 a‐OTf** to isoindol‐2‐ium chlorides on a large scale utilizing only simple and inexpensive reagents available in every laboratory. The molecular structure of the cation and the presence of the chloride anion in the product **1 a‐Cl** was confirmed using XRD (Figure 4). The distance between H2⋅⋅⋅Cl1^−^ atoms is 2,58 Å and the angle C2−H2⋅⋅⋅Cl1^−^ of 144° is in line with previously reported structures of molecules featuring C−H⋅⋅⋅Cl^−^ hydrogen bonds.^20^ The enhanced acidity of the iminium proton of **1 a‐Cl** in comparison to **1 a‐OTf** should be due to presence of such C2−H2⋅⋅⋅Cl1^−^ hydrogen bond and can also be deduced from the ^1^H NMR spectra of these compounds recorded in CDCl_3_, in which the resonances of these C−H protons differ by almost 1 ppm (*δ*=10.32 in **1 a‐OTf** vs. 11.29 ppm in **1 a‐Cl**, see Figure S32).20a

**Scheme 6:**
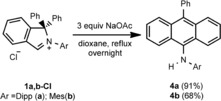
Synthesis of **4 a** and **4 b**.

To deprotonate the iminium salts, we used less (compared to alkoxides, silazanes and others) nucleophilic yet basic metal acetates. The reaction of **1 a**,**b‐Cl** with a threefold excess of sodium acetate in refluxing dioxane resulted indeed in the formation of rearrangement products **4 a**,**b** in 91 and 68 % yield, respectively (Scheme 6). The compounds **4 a**,**b** were characterized by NMR spectroscopy and HRMS analysis, and the analytical data clearly reveal that the reaction products were not the corresponding cAArCs but rearrangement products thereof (Scheme 6). The ^1^H NMR spectrum of **4 a** reveals a characteristic singlet at *δ*=6.04 ppm, which was assigned to an amine proton (no cross signal observed in the ^1^H‐^13^C HSQC), a doublet observed at *δ*=1.00 ppm was assigned to four equivalent methyl groups and sets of characteristic signals in the aromatic region were indicative for a symmetric anthracene core.

**Figure 5:**
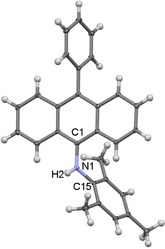
Molecular structure of **4 b** in the solid state (ellipsoids set at the 33 % probability level). Selected bond lengths [Å] and angles [°]: N1−C1 1.4258 (19), N1−H2 0.86 (2), N1−C15 1.4092 (19); C1‐N1‐C15 125.27 (12), C1‐N1‐H2 113 (1), H2‐N1‐C15 117 (1).

The diarylamines **4 a**,**b** were isolated as bright yellow, air sensitive powders, which should be stored under an argon atmosphere. Both compounds decompose slowly in the presence of air, and decomposition can be easily followed by a color change from bright to brownish yellow. The synthesis of both compounds can be carried out at gram scale, delivering a pure product in high yield in the case of **4 a**, whereas the synthesis of **4 b** leads to the formation of small amounts of so far unidentified impurities. However, the latter can be removed by washing with hexane, although the additional purification steps necessary result in a lower isolated yield. The slow diffusion of hexane into a saturated solution of **4 b** in diethyl ether at room temperature led to the formation of single crystals of this compound suitable for XRD analysis (Figure 5).

**Scheme 7:**
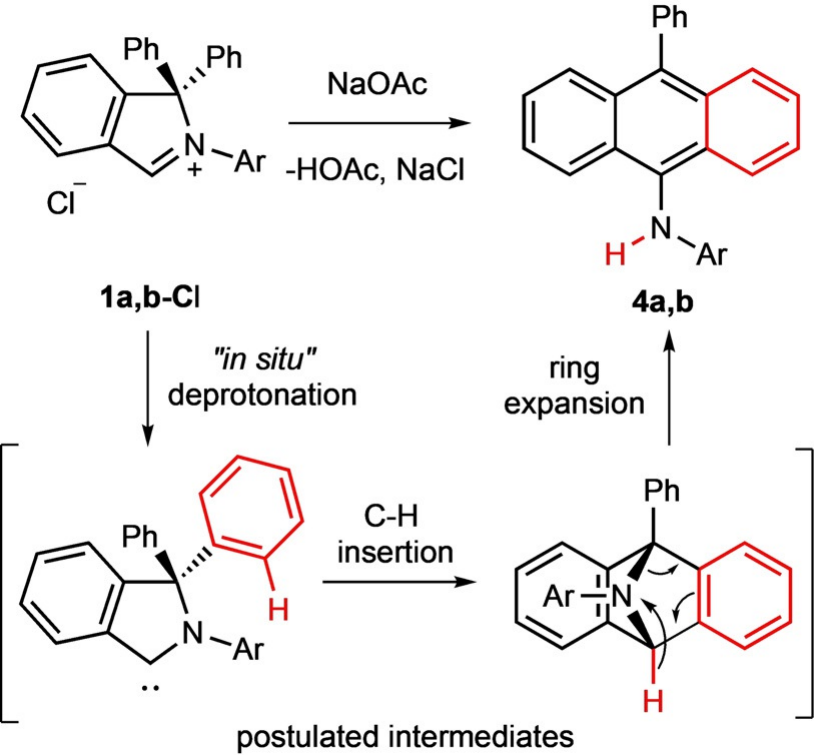
The proposed mechanism of formation of **4 a**,**b**.

The molecular structure of **4 b** confirms that the neutral anthracene derivative 9‐N(H)(Dipp)‐10‐Ph‐C_14_H_8_ has been formed by deprotonation of **1 b‐Cl** and thus the formation of a rearrangement product starting from the cAArC precursor. Formation of **4 a**,**b** presumably follows the pathway presented in Scheme 7. That is, insertion of an *in situ* formed free cAArC into the C−H bond at the *ortho* position of one of the phenyl rings of the isoindoline backbone followed by ring expansion of the five‐membered ring accompanied by aromatization of the newly‐formed six‐membered ring (Scheme 7). Ring‐expansion reaction (RER) has been reported earlier for NHCs and CAACs,^21^ although they usually proceed by intermolecular insertion towards the H−X bond (X=Si, B, Al, Sn) followed by incorporation of the heteroatom into the new rings. In contrast, the RER of 1,1‐diphenylisoindoline‐3‐ylidenes is intramolecular and includes the insertion into a C−H bond that resulted in the integration of a carbon atom into the expanded ring. Interestingly, both the intra‐ and intermolecular C−H activation have been observed before using related cAACs, albeit did not lead to any RER products even at elevated temperatures.^22^ The selectivity of the carbene insertion may be rationalized by the geometry of the C−H activation step. The molecular structure of **1 a‐Cl** in the solid state already shows that the *ortho* C−H position of the phenyl group is one of the closest C−H bonds to the carbenic carbon atom. Although a methine proton of an isopropyl substituent would be slightly closer to the *in situ* formed carbene, the reaction at this position is probably unfavored due to the restricted rotation of the aryl group attached to the nitrogen atom around the N1−C22 bond. (Figure 4).

**Scheme 8:**
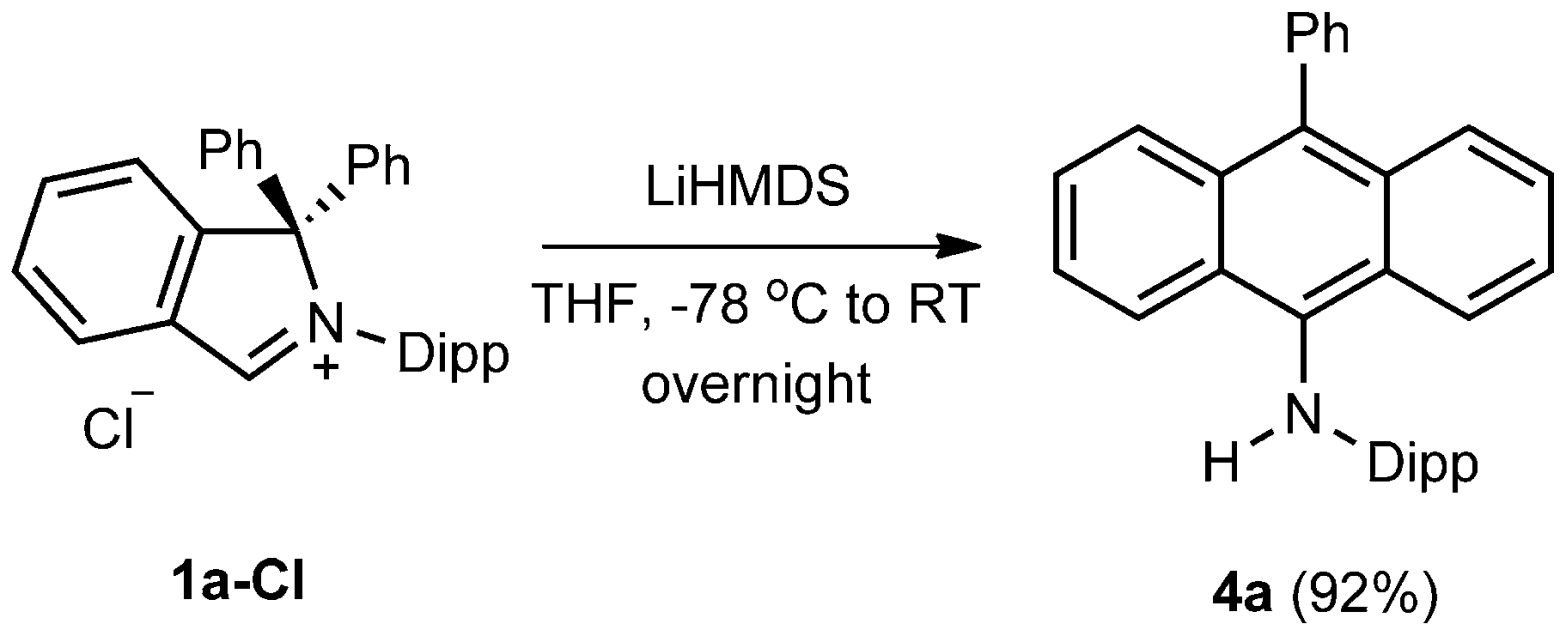
Formation of **4 a** by deprotonation of **1 a‐Cl** with LiHMDS.

To receive further support for the RER proposed here, we investigated the formation of **4 a** using isoindoline‐3‐ylidene generation conditions as proposed by Zeng and Bertrand. It has been reported previously that the attempts of low‐temperature deprotonation of **1 a‐OTf** with MN(SiMe_3_)_2_ (M=K, Na, Li), KO*t*Bu, and NaH resulted in a complex mixture of products.^9^ In sharp contrast, the reaction of isoindol‐2‐ium chloride **1 a‐Cl** with LiHMDS in THF at −78 °C resulted in the almost quantitative formation of **4 a**, which was isolated in 92 % from a crude reaction mixture by extraction with toluene (Scheme 8). This result further supports the involvement of *in situ* formed cAArC in the process of the formation of **4 a** and also indicates that this kind of RER can proceed even at low temperatures. Noteworthy, it is an impressive example that the right choice of the anion is crucial in this kind of chemistry, the chloride anion seems to facilitate the deprotonation process compared to the triflate and leads to the almost quantitative formation of **4 a**. Notably, a similar counterion effect allowed the first isolation of an abnormal NHC (aNHC), and the exchange of a tetrafluoroborate ion with the smaller halide anions was the key step required for a clean iminium salt deprotonation.^23^

**Scheme 9:**
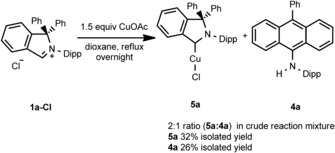
The reaction of **1 a‐Cl** with copper(I) acetate.

All attempts to spectroscopically characterize or isolate the cAArC formed *in situ* or any reaction intermediate have failed so far. However, definitive proof of 1,1‐diphenylisoindoline‐3‐ylidenes formation under acetate deprotonation conditions was obtained by a successful cAArC entrapping into the copper(I) coordination sphere. The reaction of **1 a‐Cl** with 1.5 equivalents of copper(I) acetate^24^ in refluxing dioxane results in the formation of the desired cAArC copper(I) complex **5 a**, but also the formation of the RER product **4 a** was observed in a significant amount (Scheme 9). After filtration and evaporation, a mixture of **5 a** and **4 a** in a ratio of approximately 2 to 1 was observed according to ^1^H NMR spectroscopy (Scheme 6 and Figure S30).

**Figure 6:**
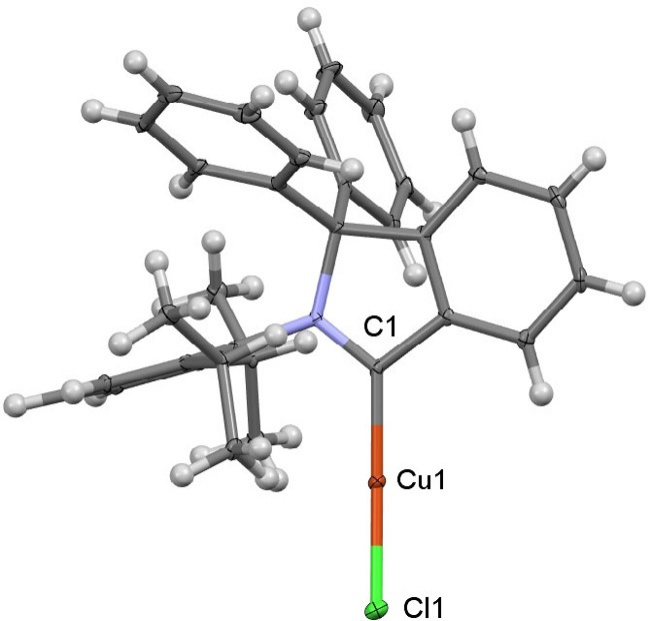
Molecular structure of **5 a** in the solid state (ellipsoids set at the 33 % probability level). Selected bond lengths and angles are presented in Table 1.

The separation of the reaction products was achieved by extraction of the crude mixture with hexane and filtration, **4 a** was isolated in the hexane fraction in 26 % yield. Subsequent extraction with dichloromethane of the material not soluble in hexane led to the isolation of **5 a** in 32 % yield. Copper complex **5 a** was characterized using ^1^H and ^13^C{^1^H} NMR spectroscopy, as well as high‐resolution mass spectrometry and X‐ray analysis. The C_carbene_−Cu resonance was found at *δ*=231.3 ppm (CDCl_3_) in the ^13^C{^1^H} NMR spectrum of **5 a**. The molecular structure in the solid state reveals that **5 a** is a linear copper complex, two‐coordinated with the cAArC and the chloride ligand. The C_carbene_‐Cu‐Cl angle is 173.35° and the Cu−C_carbene_ distance is 1.879 Å (Figure 6), which compares well with similar copper complexes bearing different types of carbene ligands (Table 1). Similarly, the cAArC carbene carbon resonance of [(cAArC)CuCl] **5 a** compares well with the NMR shifts of related cAAC complexes [(cAAC)CuCl] in the ^13^C{^1^H} NMR spectrum of these compounds.

**Table 1:** Comparison of selected bond lengths [Å] and angles [°] (esd) and C_C−Cu_ shifts on ^13^C NMR spectra in CDCl_3_ (ppm) of carbene−Cu−Cl complexes supported with various types of N‐heterocyclic carbene ligands.^26^

Ligand type	cAArC	aNHC	cAAC	MIC	NHC
Cu1−Cl bond	1.879 (3)	1.871 (7)	1.895 (6)	1.876 (4)	1.881 (7)
Cl1‐Cu1‐C1 angle	173.35 (9)	173.53 (17)	174.18 (17)	174.84 (17)	176.65 (2)
*δ* C_C−Cu_ shifts	231.3	159.5	248.5	166.4	181.0

Compound **5 a** is, to the best to our knowledge, the first example of a structurally characterized, cAArC‐supported copper complex. Compared to other carbene copper complexes,^25^ [(cAArC)CuCl] **5 a** is highly unstable in the presence of traces of air and moisture. Decomposition of **5 a** leads to a complex mixture of products and the formation of an insoluble green precipitate, which has not been identified so far. However, **5 a** can be stored as a solid in an argon filled glove‐box for a longer period of time and no decomposition was observed after three months. Notably, the gold analogue of **5 a** has been reported before and prepared only in slightly better 46 % isolated yield. Experiments to use **5 a** as a cAArC‐transfer reagent or catalyst are underway.

## Conclusions

The current study presents the investigations aiming at the generation of free isoindoline‐3‐ylidenes (cAArCs) using different starting materials. Different 3‐alkoxyisoindolines **2 a**,**b‐OR** were prepared in very good yields by treatment of alcohol solutions of 2‐aryl‐1,1‐diphenylisoindol‐2‐ium triflates **1 a**,**b‐OTf** (**a**: aryl=2,6‐di*iso*propyl‐phenyl; **b**: 2,4,6‐trimethyl‐phenyl; R=Me, Et, *i*Pr) with NEt_3_ at room temperature. The use of these indolines as cAArC precursors has been evaluated. DTA/TG analysis on solid‐state material reveals that these compounds lose alcohol by a high temperature α‐elimination process, but all attempts to synthesize the cAArCs from heating the hemiaminal ethers **2 a**,**b‐OR** under dynamic vacuum led to the formation of the rearrangement product **4** and/or to the decomposition of the material. In solution, full conversion to the cAArC was not achieved due to an equilibrium between HOR α‐elimination from the hemiaminal ether and HOR addition to the carbene cAArC. However, longer reaction times also led to the formation of **4**, and refluxing **2 a‐OMe** in toluene in the presence of elemental sulfur resulted in the high‐yield formation of the thioamide **3 a‐S**. For both reactions, the formation of traces of the free cAArC in solution is assumed. Starting with **1 a**,**b‐OTf** a high‐yielding synthesis of isoindol‐2‐ium chlorides **1 a**,**b‐Cl** was developed, which includes the synthesis of **2 a**,**b‐OR** and the subsequent treatment of **2 a**,**b‐OR** with an excess of hydrochloric acid. The reaction of **1 a**,**b‐Cl** with an excess of sodium acetate in refluxing dioxane led to the formation of rearrangement products of the cAArC, that is, the anthracene derivatives **4 a**,**b** in high yields. Compound **4 b** was structurally characterized, and the molecular structure of **4 b** confirms the connectivity of the amine and thus the formation of the rearrangement product starting from the cAArC precursor. The reaction of the isoindol‐2‐ium chloride **1 a‐Cl** with the much stronger base LiHMDS in THF led, already at −78 °C, to the formation of **4 a** in excellent yield. Thus, rearrangement of the cAArC to give the product of a ring expansion **4 a**,**b** already takes place at low temperatures and is thus associated with a small energy barrier. This is most probably the reason why all attempts to spectroscopically characterize or isolate the cAArC formed *in situ* by deprotonation or any other reaction intermediate have failed so far. However, it was possible to trap the cAArC formed at low temperatures in a copper(I) complex starting from **1 a‐Cl** and copper(I) acetate as both copper source and the base. This reaction afforded mixtures of the cAArC copper(I) complex [(cAArC)CuCl] **5 a** and RER product **4 a** in a ratio of about 2 to 1. This clearly demonstrates that deprotonation of isoindol‐2‐ium chlorides with base leads to the formation of the cAArC, which rearranges rather quickly, even in the presence of a trapping reagent, to the RER product **4 a**.

## Experimental Section

### General methods and chemicals

All reactions and subsequent manipulations were performed under an argon atmosphere in an Innovative Technology Inc. glovebox or using standard Schlenk techniques, if not stated otherwise. ^1^H and ^13^C{^1^H} NMR spectra were recorded on a Varian 400 or Bruker Avance 400 at 25 °C. ^1^H NMR chemical shifts are reported relative to TMS (*δ* in ppm) and were referenced by residual proton resonances of the corresponding deuterated solvent (CHCl_3_: 7.26 ppm; C_6_D_5_H: 7.16 ppm) whereas ^13^C{^1^H} NMR spectra are reported relative to TMS using the natural‐abundance carbon resonances (CDCl_3_: 77.16 ppm; C_6_D_6_: 128.06 ppm). Coupling constants are given in Hertz. Mass spectrometry (EI+) analyses were performed using magnetic sector mass spectrometer AutoSpec Premier (Waters, USA), equipped with an electron impact (EI) ion source and the EBE double focusing geometry mass analyzer. The electron energy was set to 70 eV. ESI(+) mass spectrometry analyses were performed using Synapt G2‐S mass spectrometer (Waters) equipped with the Electrospray ion source and quadrupole‐time‐of‐flight mass analyzer. Dichloromethane was used as a solvent. The measurement was performed in positive ion mode with the desolvation gas flow 600 L h^−1^ and capillary voltage set to 4500 V with the flow rate 100 μL min^−1^. TGA analyses were performed using a TGA4000 (PerkinElmer) thermal gravimetric analyzer. The measurements were conducted in a nitrogen atmosphere (flow of 20 cm^3^ min^−1^), in a temperature range of 25–600 °C, and heating rate 10 °C min^−1^. The chemicals were obtained from Aldrich (including copper(I) acetate) except for potassium hydroxide and ethanol 99.8 % purchased from POCH S.A. BASIC (Poland). The compounds **1a,b‐OTf** were prepared according to the literature.^[S1]^ All other solvents for synthetic reactions were HPLC grade further treated to remove traces of water using an Innovative Technology Inc. Pure‐Solv Solvent Purification System and deoxygenated using the freeze‐pump‐thaw method.

### Synthetic procedures

#### Preparation and characterization of isoindol‐2‐ium chlorides 1 a,b‐Cl

In a 100 mL baker, **2 a**,**b‐OMe** (1 mmol) was dissolved in 10 mL of Et_2_O. Water (10 mL) was added to the solution and then a tenfold excess of hydrochloric acid (water solution) was dropped to the mixture under vigorous stirring. After addition of the acid the immediate formation of a precipitate was observed, and the reaction mixture was stirred for another 15 minutes. The precipitate was filtered off, washed with a diluted solution of the hydrochloric acid (few drops of concentrated acid in 20 mL of water) and diethyl ether (2×10 mL). The residue was dried in vacuo to give **1 a**,**b‐Cl** in the form of a colorless crystalline powder.

**1 a‐Cl**: white crystalline powder (438 mg, 94 %). Single‐crystals suitable for X‐ray diffraction were obtained by slow evaporation of a saturated DCM solution of **1 a‐Cl** at room temperature. ^1^H NMR (400 MHz, 25 °C, CDCl_3_): *δ*=11.14 (s, 1 H, CH_im_), 9.14 (d, *J=*7.7 Hz, 1 H, CH_Ar_), 7.86 (td, *J=*7.6, 1.2 Hz, 1 H, CH_Ar_), 7.80 (td, *J=*7.8, 0.8 Hz, 1 H, CH_Ar_), 7.42–7.36 (m, 4 H, CH_Ar_), 7.30–7.26 (m, 4 H, overlapping with residue solvent signal, CH_Ar_), 7.21 (d, *J=*7.6 Hz, 1 H, CH_Ar_), 7.11–7.07 (m, 6 H, CH_Ar_), 2.17 (hept, *J=*6.8 Hz, 2 H, CH_*i*Pr_), 1.11 (d, *J=*6.8 Hz, 6 H, CH_3*i*Pr_), 0.20 ppm (d, *J=*6.6 Hz, 6 H, CH_3*i*Pr_).^13^C{^1^H} NMR (100 MHz, 25 °C, CDCl_3_): *δ*=154.9 (CH_im_), 146.8, 136.9, 133.4, 133.21, 132.2, 132.1, 131.8, 131.3, 130.7, 130.6, 130.5, 130.3, 128.9, 125.7, 125.3, 95.3 (CPh_2_), 30.6 (CH_*i*Pr_), 26.7 (CH_3*i*Pr_), 21.9 ppm (CH_3*i*Pr_)., HRMS: calc. for [*M*−Cl^−^] C_32_H_32_N *m*/*z*: 430.2530; found: 430.2531

**1 b‐Cl**: white crystalline powder (387 mg, 92 %). ^1^H NMR (400 MHz, 25 °C, CDCl_3_ ): *δ*=10.60 (s, 1 H, CH_im_), 8.75 (d, *J=*7.8 Hz, 1 H, CH_Ar_), 7.89 (t, *J=*7.6 Hz, 1 H, CH_Ar_), 7.77 (t, *J=*7.6 Hz, 1 H, CH_Ar_), 7.40 (t, *J=*7.4 Hz, 2 H, CH_Ar_), 7.32–7.21 (m, 5 H, overlapping with CDCl_3_ signal, CH_Ar_), 7.08 (d, *J=*7.8 Hz, 4 H, CH_Ar_), 6.73 (s, 2 H, CH_Mes_), 2.23 (s, 3 H, *para*‐CH_3_), 1.37 ppm (s, 6 H, *ortho*‐CH_3_). ^13^C{^1^H} NMR (100 MHz, 25 °C, CDCl_3_): *δ*=178.0 (CH_im_), 155.2, 141.5, 137.4, 136.0, 133.6, 131.9, 131.3, 131.0, 130.6, 130.4, 130.3, 128.9, 125.7, 94.9 (CPh_2_), 21.00 (*para*‐CH_3_), 19.3 ppm (*ortho*‐CH_3_). HRMS: calcd *m*/*z* for [*M*−Cl^−^] C_29_H_26_N : 388.2060; found: 388.2057.

#### Preparation and characterization of 3‐(alkoxy)‐substituted isoindolines (2 a,b‐OR, R=Me, Et, iPr))

The compounds **1 a** or **1 b** (1 mmol), respectively, were suspended/dissolved under an inert atmosphere in a 25 mL Schlenk tube equipped with a magnetic stirring bar and a stopcock in anhydrous alcohol (5 mL). Anhydrous triethylamine (1.5 mmol, 1.5 equiv) was added and the reaction mixture was stirred overnight at room temperature. Afterwards, the reaction mixture was evaporated to dryness and the residue was dissolved/suspended in 2×5 mL of anhydrous Et_2_O (dry hexane can be also used), filtered and evaporated to dryness to yield the pure products as a white powders.

**2 a‐OMe**: white powder (429 mg, 93 %). Single‐crystals suitable for X‐ray diffraction were obtained by recrystallization in methanol/DCM. ^1^H NMR (400 MHz, 25 °C, CDCl_3_): *δ*=7.59–7.77 (m, 1 H, CH_Ar_), 7.41–7.39 (m, 2 H, CH_Ar_), 7.37–7.33 (m, 2 H, CH_Ar_), 7.17–7.08 (m, 9 H, CH_Ar_), 6.93–6.88 (m, 2 H, CH_Ar_), 6.80–6.78 (m, 1 H, CH_Ar_), 5.96 (s, 1 H, OCH), 3.93–3.86 (m, 1 H, CH_*i*Pr_), 3.52 (s, 3 H, OCH_3_), 2.09–2.03 (m, 1 H, CH_*i*Pr_), 1.15 (d, *J=*6.8 Hz, 1 H, CH_3*i*Pr_), 1.09 (d, *J=*6.7 Hz, 1 H, CH_3*i*Pr_), 0.47 (d, *J=*6.8 Hz, 1 H, CH_3*i*Pr_), −0.08 ppm (d, *J=*6.6 Hz, 1 H, CH_3*i*Pr_). ^13^C{^1^H} NMR (100 MHz, 25 °C, CDCl_3_): *δ*=151.4, 149.0, 148.9, 147.55, 141.8, 138.1, 137.9, 130.8, 129.3, 128.3, 127.4, 127.1, 127.1, 126.9, 126.6, 126.1, 124.2, 123.9, 102.0, 93.0, 82.2, 57.5 (OCH_3_), 28.8 (CH_*i*Pr_), 28.6 (CH_*i*Pr_), 26.3 (CH_3*i*Pr_), 24.9 (CH_3*i*Pr_), 23.1 (CH_3*i*Pr_), 23.0 ppm (CH_3*i*Pr_). ^1^H NMR (400 MHz, 25 °C, C_6_D_6_): *δ*=7.75 (d, *J=*7.6 Hz, 2 H, CH_Ar_), 7.52–7.49 (m, 1 H, CH_Ar_), 7.19 (d, *J=*2.0 Hz, 1 H, CH_Ar_), 7.16–6.99 (m, 7 H, CH_Ar_), 6.98–6.88 (m, 5 H, CH_Ar_), 6.83 (dd, *J=*7.4, 2.0 Hz, 1 H, CH_Ar_), 6.08 (s, 1 H, OCH), 4.36–4.17 (m, 1 H, CH_*i*Pr_), 3.43 (s, 3 H, OCH_3_), 2.33–2.20 (m, 1 H, CH_*i*Pr_), 1.38 (d, *J=*6.8 Hz, 3 H, CH_3*i*Pr_), 1.11 (d, *J=*6.7 Hz, 3 H, CH_3*i*Pr_), 0.76 (d, *J=*6.8 Hz, 3 H, CH_3*i*Pr_), 0.04 ppm (d, *J=*6.6 Hz, 3 H, CH_3*i*Pr_).^13^C{^1^H} NMR (100 MHz, 25 °C, C_6_D_6_): *δ*=152.0, 149.6, 149.5, 148.2, 142.2, 138.9, 138.5, 131.2, 129.9, 128.6, 127.9, 127.6, 127.6, 127.5, 127.3, 127.1, 126.6, 124.8, 124.7, 124.3, 102.5 (OCH), 82.6 (CPh_2_), 57.3 (OCH_3_), 29.3 (CH_*i*Pr_), 29.0 (CH_*i*Pr_), 26.5 (CH_3*i*Pr_), 25.5 (CH_3*i*Pr_), 23.6 (CH_3*i*Pr_), 23.4 ppm (CH_3*i*Pr_). HRMS: calcd for C_33_H_35_NO *m*/*z*: 461.2719; found: 461.2697.

**2 a‐OEt**: white powder (449 mg, 95 %). Single‐crystals suitable for X‐ray diffraction were obtained by recrystallization in ethanol/DCM. ^1^H NMR (400 MHz, 25 °C, C_6_D_6_): *δ*=7.69 (d, *J=*7.6 Hz, 1 H, CH_Ar_), 7.46–7.44 (m, 1 H, CH_Ar_), 7.12–7.10 (m, 1 H, CH_Ar_), 7.09–6.92 (m, 7 H, CH_Ar_), 6.91–6.81 (m, 5 H, CH_Ar_), 6.78–6.74 (m, 1 H, CH_Ar_), 6.14 (s, 1 H, OCH), 4.20–4.13 (m, 1 H, CH_*i*Pr_), 3.62 (dq, *J=*9.2, 7.0 Hz, 1 H, CH_3_CH_2_O), 3.52 (dq, *J=*9.2, 6.9 Hz, 1 H, CH_3_CH_2_O), 2.23–2.17 (m, 1 H, CH_*i*Pr_), 1.28 (d, *J=*6.8 Hz, 3 H, CH_3*i*Pr_), 1.14 (t, *J=*7.0 Hz, 3 H, CH_3_CH_2_O), 1.05 (d, *J=*6.7 Hz, 3 H, CH_3*i*Pr_), 0.68 (d, *J=*6.8 Hz, 3 H, CH_3*i*Pr_), −0.03 ppm (d, *J=*6.6 Hz, 3 H, CH_3*i*Pr_). ^13^C{^1^H} NMR (100 MHz, 25 °C, C_6_D_6_): *δ*=151.9, 149.8, 149.5, 148.2, 142.4, 139.0, 138.6, 129.9, 128.5, 127.6, 127.3, 127.0, 126.6, 124.8, 124.6, 124.5, 100.6 (OCH), 82.6 (CPh_2_), 65.1 (CH_3_ CH_2_O), 29.2 (CH_*i*Pr_), 29.0 (CH_*i*Pr_), 26.5 (CH_3*i*Pr_), 25.3 (CH_3*i*Pr_), 23.7 (CH_3*i*Pr_), 23.4 (CH_3*i*Pr_), 16.2 ppm (CH_3_CH_2_O). HRMS: calcd for C_34_H_37_NO *m*/*z*: 475.2875; found: 475.2796.

**2 a‐O*i*Pr**: white powder (427 mg, 87 %). Single‐crystals suitable for X‐ray diffraction were obtained by recrystallization in isopropanol/DCM. ^1^H NMR (400 MHz, 25 °C, C_6_D_6_): *δ*=7.55–7.47 (m, 1 H, CH_Ar_), 7.17–7.16 (m, overlapping with benzene, 2 H, CH_Ar_), 7.16–7.08 (m, 4 H, CH_Ar_), 7.07–6.98 (m, 4 H, CH_Ar_), 6.97–6.87 (m, 5 H, CH_Ar_), 6.81 (dd, *J=*7.5, 1.9 Hz, 1 H, CH_Ar_), 6.34 (s, 1 H, OCH), 4.25–4.15 (m, 1 H, CH_*i*Pr_), 3.92–3.79 (m, 1 H, OCH_*i*Pr_), 2.32–2.23 (m, 1 H, CH_*i*Pr_), 1.34 (d, *J=*6.9 Hz, 3 H, CH_3*i*Pr_), 1.19 (d, *J=*2.4 Hz, 3 H, OCHCH_3_ ), 1.18 (d, *J=*2.3 Hz, 3 H, OCHCH_3_), 1.13 (d, *J=*6.7 Hz, 1 H, CH_3*i*Pr_), 0.73 (d, *J=*6.8 Hz, 1 H, CH_3*i*Pr_), 0.03 ppm (d, *J=*6.6 Hz, 1 H, CH_3*i*Pr_). ^13^C{^1^H} NMR (100 MHz, 25 °C, C_6_D_6_): *δ*=152.0, 150.1, 149.6, 148.2, 142.6, 139.5, 138.9, 129.9, 128.3, 127.7, 127.7, 127.6, 127.2, 127.1, 126.6, 124.8, 124.6, 124.5, 97.6 (ONCH), 82.7 (CPh_2_), 69.2 (OCH_*i*Pr_), 29.1, 29.0, 26.4, 25.6, 23.8, 23.8, 23.6, 23.2 ppm. HRMS: calcd for C_35_H_39_NO *m*/*z*: 489.3032; found: 489.3027.

**2 b‐OMe**: white powder (384 mg, 92 %). Single‐crystals suitable for X‐ray diffraction were obtained by recrystallization in methanol/DCM. ^1^H NMR (400 MHz, 25 °C, C_6_D_6_): *δ*=7.95 (d, *J=*7.6 Hz, 2 H, CH_Ar_), 7.53–7.50 (m, 1 H, CH_Ar_), 7.15–7.08 (m, 5 H, CH_Ar_), 7.07–6.98 (m, 2 H, CH_Ar_) 6.98–6.91 (m, 3 H, CH_Ar_), 6.90–6.83 (m, 2 H, CH_Ar_), 6.70–6.71 (m, 1 H, CH_Mes_), 6.45–6.43 (m, 1 H, CH_Mes_), 5.95 (s, 1 H, OCH), 3.42 (s, 3 H, OCH_3_), 2.42 (s, 3 H, CH_3Mes_), 2.00 (s, 3 H, CH_3Mes_), 1.46 ppm (s, 3 H, CH_3Mes_). ^13^C{^1^H} NMR (100 MHz, 25 °C, C_6_D_6_): *δ*=149.5, 148.5, 143.5, 140.9, 138.9, 138.7, 138.1, 135.5, 131.3, 129.9, 129.6, 129.6, 128.8, 127.7, 127.5, 127.4, 126.7, 126.6, 126.1, 125.0, 102.2 (OCH), 83.0 (CPh_2_), 56.5 (OCH_3_), 22.9 (CH_3Mes_), 20.8 (CH_3Mes_), 19.8 ppm (CH_3Mes_). HRMS: calcd for C_30_H_29_NO *m*/*z*: 419.2249; found: 419.2263.

**2 b‐OEt**: white powder (408 mg, 94 %) ^1^H NMR (400 MHz, 25 °C, C_6_D_6_): *δ*=7.95 (d, *J=*7.7 Hz, 2 H, CH_Ar_), 7.52 (d, *J=*8.1 Hz, 1 H, CH_Ar_), 7.15–7.07 (m, 6 H, CH_Ar_), 7.06–6.98 (m, 2 H, CH_Ar_), 6.98–6.92 (m, 3 H, CH_Ar_), 6.90–6.83 (m, 2 H, CH_Ar_), 6.72 (s, 1H CH_Mes_), 6.45 (s, 1 H, CH_Mes_), 6.07 (s, 1 H, OCH), 3.84 (dq, *J=*9.3, 7.0 Hz, 1 H, OCH_2_), 3.45 (dq, *J=*9.3, 7.0 Hz, 1 H, OCH_2_), 2.42 (s, 3 H, CH_3Mes_), 2.01 (s, 3 H, CH_3Mes_), 1.46 (s, 3 H, CH_3Mes_), 1.23 ppm (t, *J=*7.0 Hz, 3 H, OCH_2_CH_3_). ^13^C{^1^H} NMR (100 MHz, 25 °C, C_6_D_6_): *δ*=149.7, 148.4, 143.4, 141.0, 139.0, 139.0, 138.3, 135.5, 131.3, 129.9, 129.8, 129.6, 128.7, 127.7, 127.5, 127.4, 126.7, 126.6, 126.2, 125.1, 100.6 (OCH), 83.0 (CPh_2_), 64.7 (OCH_2_), 23.0 (CH_3Mes_), 20.8 (CH_3Mes_), 19.7 (CH_3Mes_), 16.2 ppm (OCH_2_ CH_3_). HRMS: calcd for C_31_H_31_NO *m*/*z*: 433.2406; found: 433.2398.

**2 b‐O*i*Pr**: white powder (398 mg, 89 %), ^1^H NMR (400 MHz, 25 °C, CDCl_3_): *δ*=7.48–7.46 (m, 1 H, CH_Ar_), 7.38–7.24 (m, 3 H, CH_Ar_), 7.22–7.08 (m, 5 H, CH_Ar_), 7.03–6.89 (m, 5 H, CH_Ar_), 6.76–6.62 (m, 1 H, CH_Ar,_ CH_Mes_), 6.47–6.33 (m, 1 H, CH_Mes_), 6.04 (s, 1 H, OCH), 3.81 (hept, *J=*6.1 Hz, 1 H, OCH(CH_3_)_2_), 2.18 (s, 3 H, CH_3Mes_), 2.13 (s, 3 H, CH_3Mes_), 1.35 (s, 3 H, CH_3Mes_), 1.27 (d, *J=*6.3 Hz, 3 H, CH_3*i*Pr_), 1.19 ppm (d, *J=*5.9 Hz, 3 H, CH_3*i*Pr_). ^13^C{^1^H} NMR (100 MHz, 25 °C, CDCl_3_): *δ*=149.3, 147.9, 142.8, 140.9, 138.8, 138.7, 138.0, 135.3, 130.9, 129.4, 129.3, 128.9, 128.5, 127.7, 127.3, 127.0, 126.4, 126.1, 125.9, 124.4, 97.3, (NCH) 82.4 (CPh_2_), 69.6 (OCH_*i*Pr_), 23.9, 22.6, 22.6, 20.8, 19.6 ppm. HRMS: calcd for C_32_H_33_NO *m*/*z*: 447.2562; found: 447.2569.

#### Preparation and characterization of thioamide 3 a

A 25 mL Schlenk tube equipped with a stirring bar and Rotaflo stopcock was charged with **2 a** (100 mg, 0.17 mmol) and sulphur (11.1 mg, 0.35 mmol, 2.00 equiv), 5 mL of dry toluene and the reaction mixture was refluxed at 130 °C in a closed vessel overnight. Afterwards, all volatile material was removed under reduced pressure and the thioamide **3 a** was purified by column chromatography on silica gel **(**hexane/EtOAc 50:1) to give 74 mg of a pale‐yellow solid (92 % yield). ^1^H NMR (400 MHz, 25 °C, CDCl_3_): *δ*=8.34–8.27 (m, 1 H, CH_Ar_), 7.61–7.51 (m, 2 H, CH_Ar_), 7.33–7.29 (m, 1 H, CH_Ar_), 7.25–7.14 (m, 7 H, CH_Ar_), 7.12–7.07 (m, 1 H, CH_Ar_), 7.02 (dd, *J=*15.7, 7.5 Hz, 6 H, CH_Ar_), 2.42–2.35 (m, 1 H, CH_*i*Pr_), 1.06 (d, *J=*6.7 Hz, 1 H, CH_3*i*Pr_), 0.06 ppm (d, *J=*6.7 Hz, 1 H, CH_3*i*Pr_). ^13^C{^1^H} NMR (100 MHz, 25 °C, CDCl_3_): *δ*=197.6 (C=S), 149.5, 148.34, 137.4, 137.00, 135.3, 132.0, 130.3, 129.3, 128.6, 128.2, 127.8, 126.6, 125.4, 124.5, 86.1 (CPh_2_), 30.1 (CH_*i*Pr_), 26.0 (CH_3*i*Pr_), 22.1 ppm (CH_3*i*Pr_). ^1^H and ^13^C NMR spectra matched those reported in the literature.^9^

#### Preparation and characterization of diarylamines 4 a,b

**Compound 4 a**: A 100 mL Schelenk flask equipped with a magnetic stirring bar and a stopcock was charged with **1 a‐Cl** (1.00 g, 1.72 mmol) and sodium acetate (530 mg, 5.16 mmol, 3.00 equiv) and 20 mL of anhydrous dioxane was added. The reaction mixture was stirred overnight at 120 °C and then the solvent was removed under reduced pressure. The residue was taken up with 25 mL of dry hexane, insoluble particles were filtered through a pad of celite, which was washed with dry hexane until all yellow color was removed. The filtrate was then evaporated to dryness to get pure **4 a** as an air sensitive yellow powder (836 mg, 91 %). ^1^H NMR (400 MHz, 25 °C, C_6_D_6_): *δ*=8.21–8.05 (m, 2 H, CH_Anthr_), 7.91–7.78 (m, 2 H, CH_Anthr_), 7.45–7.35 (m, 2, CH_Ar_), 7.35–7.24 (m, 3 H, CH_Ar_), 7.22–7.17 (m, 3 H, CH_Ar_), 7.12–7.01 (m, 4 H, CH_Anthr_), 6.04 (s, 1 H, NH), 3.21 (hept, *J=*6.8 Hz, 2 H, CH_*i*Pr_), 1.00 ppm (d, *J=*6.8 Hz, 12 H, CH_3*i*Pr_). ^13^C{^1^H} NMR (100 MHz, 25 °C, C_6_D_6_): *δ*=142.1, 140.6, 140.1, 138.3, 132.3, 131.7, 130.9, 128.8, 127.4, 125.6, 125.1, 124.7, 124.4, 123.5, 123.4, 28.6, 23.6 ppm. HRMS: calc for C_32_H_31_N *m*/*z*: 429.2457; found: 429.2451.

**Compound 4 b**: A 100 mL Schlenk flask equipped with a magnetic stirring bar and a stopcock was charged with **1 b‐Cl** (100 mg, 0.24 mmol), sodium acetate (83.0 mg, 0.72 mmol, 3.00 equiv) and 7.00 mL of anhydrous dioxane. The reaction mixture was stirred overnight at 120 °C and the solvent was removed under reduced pressure afterwards. The residue was taken up with 10 mL of dry hexane and filtered through a wide pad of celite. Celite was washed with dry hexane until the filtrate was no longer red. Then, the receiver Schlenk flask was exchanged for a new one and the pad of celite was washed with dry Et_2_O until all yellow color was removed. Diethyl ether fraction was then evaporated to get pure **4 b** as an air sensitive yellow powder (61.0 mg, 68 %). Single‐crystals suitable for X‐ray diffraction were obtained by slow diffusion of hexane into a saturated solution of Et_2_O at room temperature. ^1^H NMR (400 MHz, 25 °C, C_6_D_6_): *δ*=8.18–7.99 (m, 2 H, CH_Anthr_), 7.88–7.80 (m, 2 H, CH_Anthr_), 7.40–7.37 (m, 2 H, CH_Ph_), 7.35–7.24 (m, 3 H, CH_Ph_), 7.13–7.04 (m, 4 H, CH_Anthr_), 6.79 (s, 2 H, CH_Mes_), 5.52 (s, 1 H, NH), 2.19 (s, 3 H, *para*‐CH_3Mes_), 1.91 ppm (s, 6 H, *ortho*‐CH_3Mes_). ^13^C{^1^H} NMR (100 MHz, 25 °C, C_6_D_6_): *δ*=141.3, 140.1, 137.4, 132.2, 132.2, 131.5, 131.3, 130.5, 129.2, 128.7, 127.9, 127.5, 125.6, 125.5, 124.7, 123.6, 20.8, 19.3 ppm. HRMS: calc for C_29_H_25_N *m*/*z*: 387.1987; found 387.1982.

#### Preparation and characterization of the Cu‐CAArC complex 5 a

A 100 mL Schlenk flask equipped with a magnetic stirring bar and a stopcock was charged with **1 a‐Cl** (500 mg, 1.07 mmol), copper(I) acetate (200 mg, 5.35 mmol, 5.00 equiv), and 20 mL of anhydrous dioxane. The reaction mixture was stirred overnight at 120 °C, cooled down to room temperature and filtered through a pad of Celite (residue was washed with 10 mL of dioxane). The filtrate was evaporated to dryness and the residue was taken up with 20 mL of dry hexane. The undissolved components were filtered off and washed with dry hexane (3×5 mL), and evaporated to dryness. The remaining solid was then extracted with dry dichloromethane and after evaporation of the solvent under reduced pressure the copper complex **5 a** was obtained as yellowish powder (182 mg, 32 %). Crystals suitable for X‐ray diffraction were obtained by slow diffusion of hexane into DCM solution of **5 a**. ^1^H NMR (400 MHz, 25 °C, CDCl_3_): *δ*=8.52–8.44 (m, 1 H), 7.68–7.59 (m, 2 H), 7.33 (t, *J=*7.8 Hz, 1 H), 7.31–7.26 (m, 2 H), 7.23–7.17 (m, 4 H), 7.11–7.07 (m, 1 H), 7.04 (d, *J=*7.8 Hz, 2 H), 6.94–6.89 (m, 4 H), 2.28 (hept, *J=*6.7 Hz, 2 H), 1.14 (d, *J=*6.8 Hz, 7 H), 0.13 ppm (d, *J=*6.7 Hz, 7 H). ^13^C{^1^H} NMR (100 MHz, 25 °C, CDCl_3_): *δ*=231.4, 151.2, 146.6, 143.0, 137.4, 134.1, 133.9, 132.1, 130.4, 130.3, 129.4, 129.1, 128.3, 125.5, 124.8, 95.1, 30.0, 27.2, 21.5 ppm. HRMS: calcd for [*M*−Cl]^+^ C_32_H_31_NCu *m*/*z*: 492.1753; found: 492.1747.

### X‐ray crystallographic data

CCDC 1857165 (**1a‐Cl**), 1857166 (**2a‐OMe**), 1857167 (**2a‐OEt**), 1857164 (**2a‐O*i*Pr**), 1857168 (**2b‐OMe**), 1921589 (**4b**), and 1921590 (**5a**) contain the supplementary crystallographic data for this paper. These data are provided free of charge by The Cambridge Crystallographic Data Centre.

## Conflict of interest

The authors declare no conflict of interest.

## Supporting information

As a service to our authors and readers, this journal provides supporting information supplied by the authors. Such materials are peer reviewed and may be re‐organized for online delivery, but are not copy‐edited or typeset. Technical support issues arising from supporting information (other than missing files) should be addressed to the authors.

Supplementary
