# Supplementary material for: Intramolecular Ring‐Expansion Reaction (RER) and Intermolecular Coordination of In Situ Generated Cyclic (Amino)(aryl)carbenes (cAArCs)
Source: Chemistry. 2019 Aug 2;25(48):11365–74. doi: 10.1002/chem.201902630 (PMC6771577; doi:10.1002/chem.201902630)
Supplement: Supplementary file 1 — Supplementary [file CHEM-25-11365-s001.pdf]

CHEMISTRY
A European Journal

Supporting Information

Intramolecular Ring-Expansion Reaction (RER) and Intermolecular Coordination of In Situ Generated Cyclic (Amino)(aryl)carbenes (cAArCs)

Jan Lorkowski,[a, b] Mirjam Krahfuß,[b] Maciej Kubicki,[a] Udo Radius,*[b] and Cezary Pietraszuk*[a]

chem_201902630_sm_miscellaneous_information.pdf

Table of Content

1) NMR Spectra (all recorded at 25 ^{o}C) 2
2) Additional experiments and Figures 16
3) X-ray crystallography 20
4) References 22

1) NMR Spectra (all recorded at 25 °C)

Figure S1. ^{1}H NMR spectra of **1a-OTf** ($CDCl_3$)

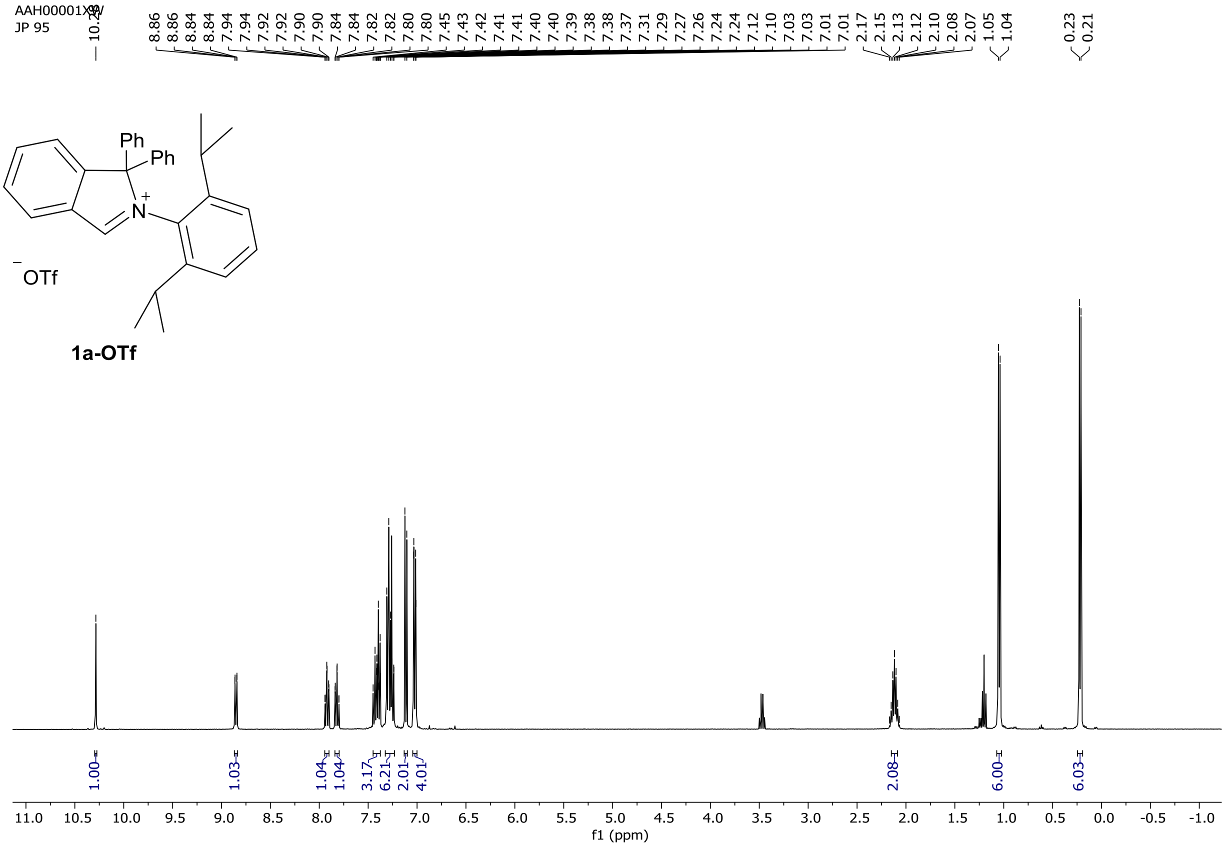

Figure S2. ^{1}H NMR spectra of **1b-OTf** ($CDCl_3$)

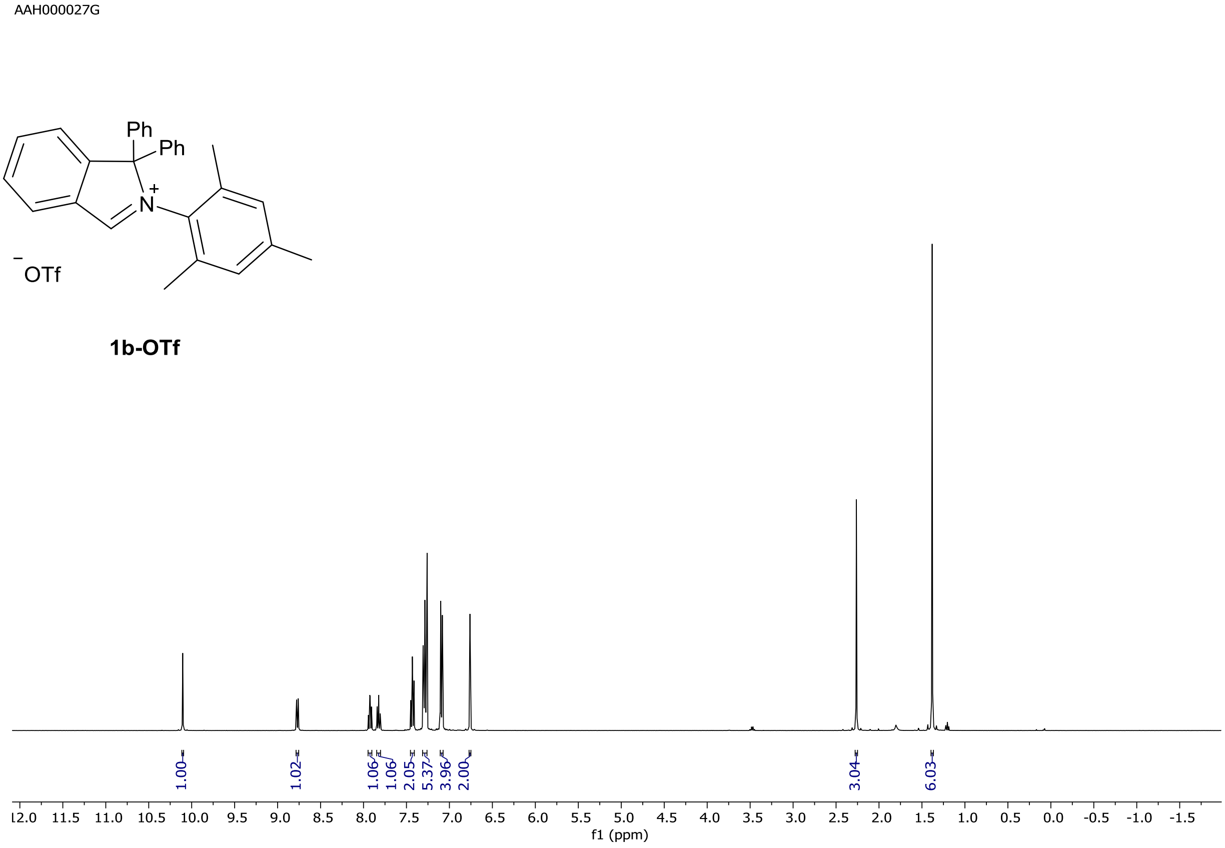

Figure S3. ^{1}H NMR spectra of **1a-Cl** ($CDCl_3$)

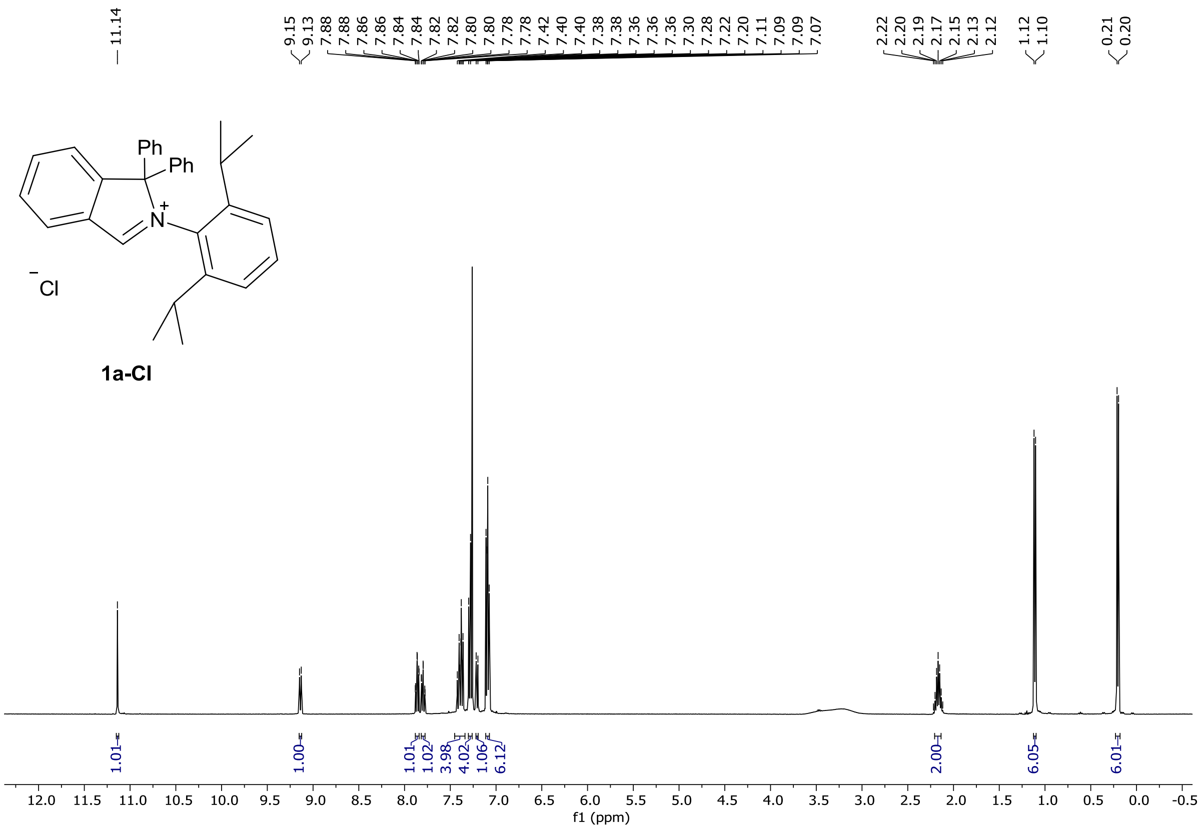

Figure S4. ^{13}C{^{1}H} NMR spectra of **1a-Cl** ($CDCl_3$)

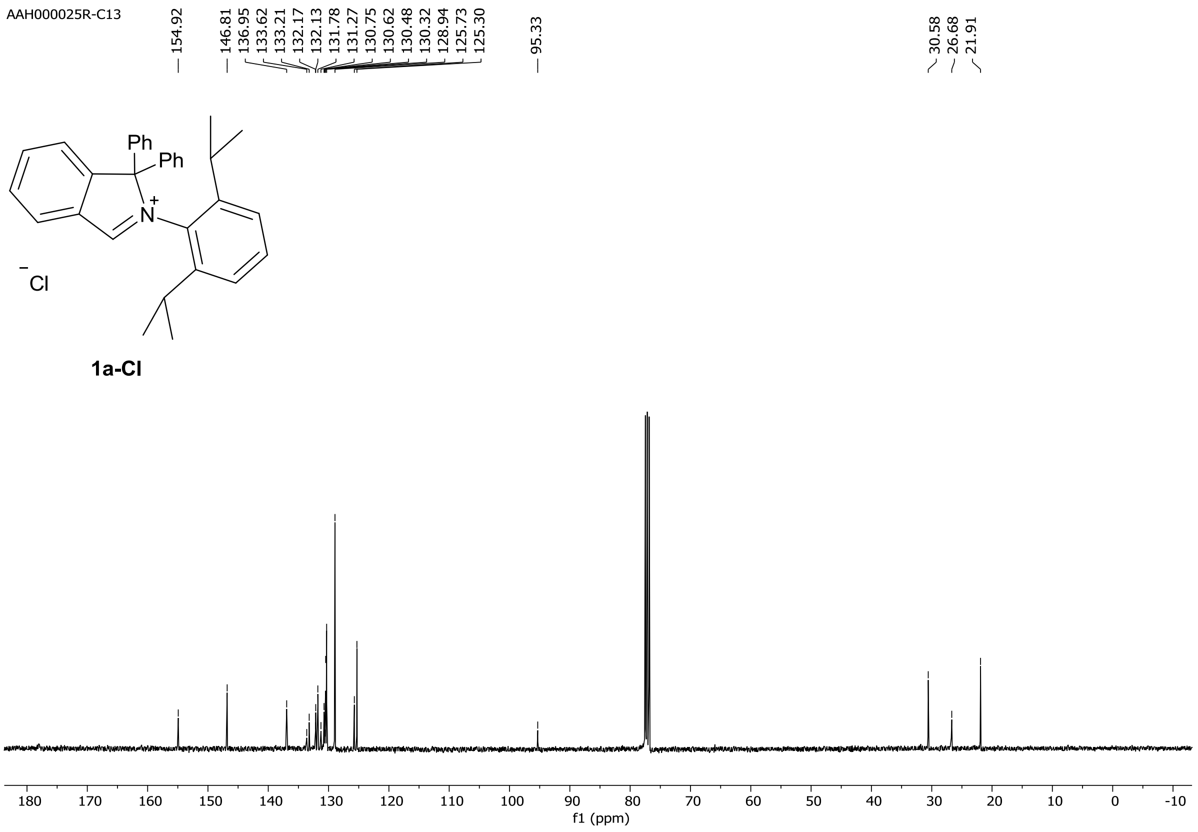

Figure S5. ^{1}H NMR spectra of **1b-Cl** ($CDCl_3$)

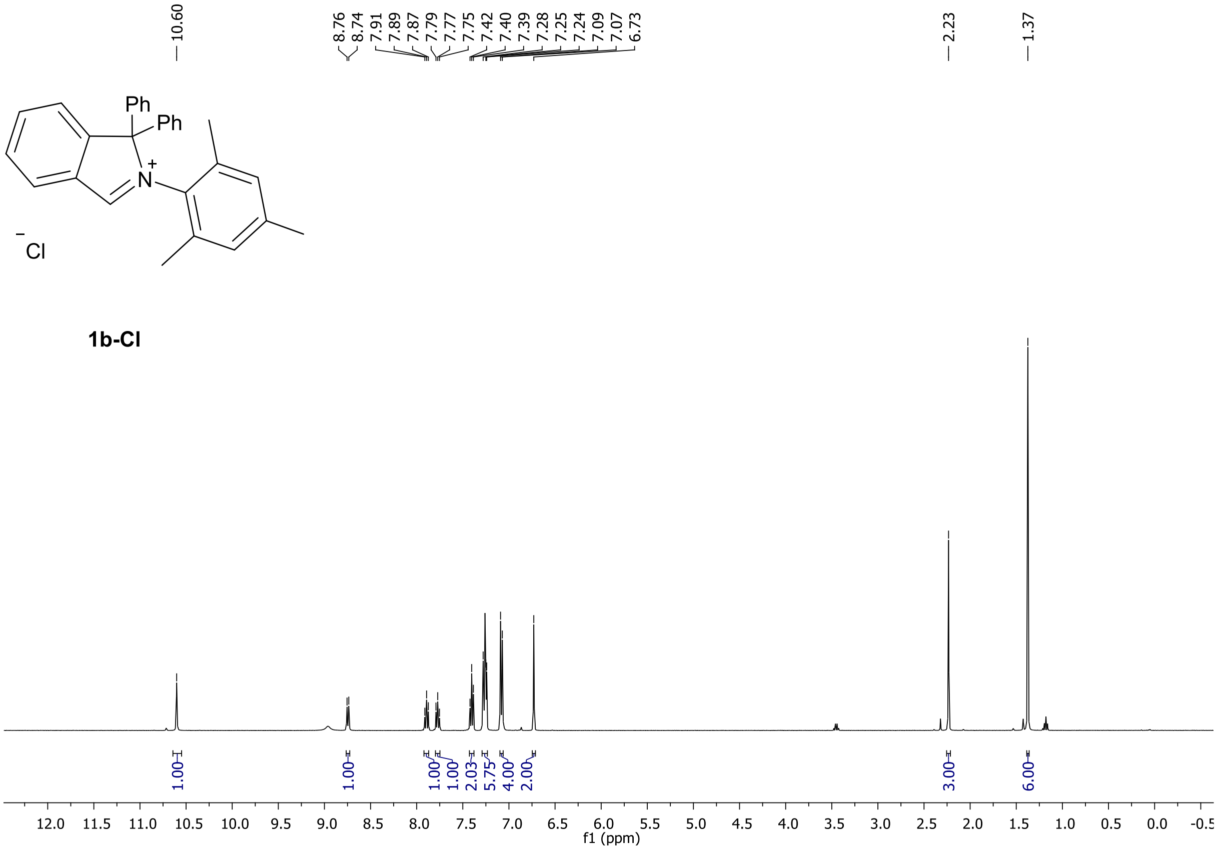

Figure S6. ^{13}C{^{1}H} NMR spectra of **1b-Cl** ($CDCl_3$)

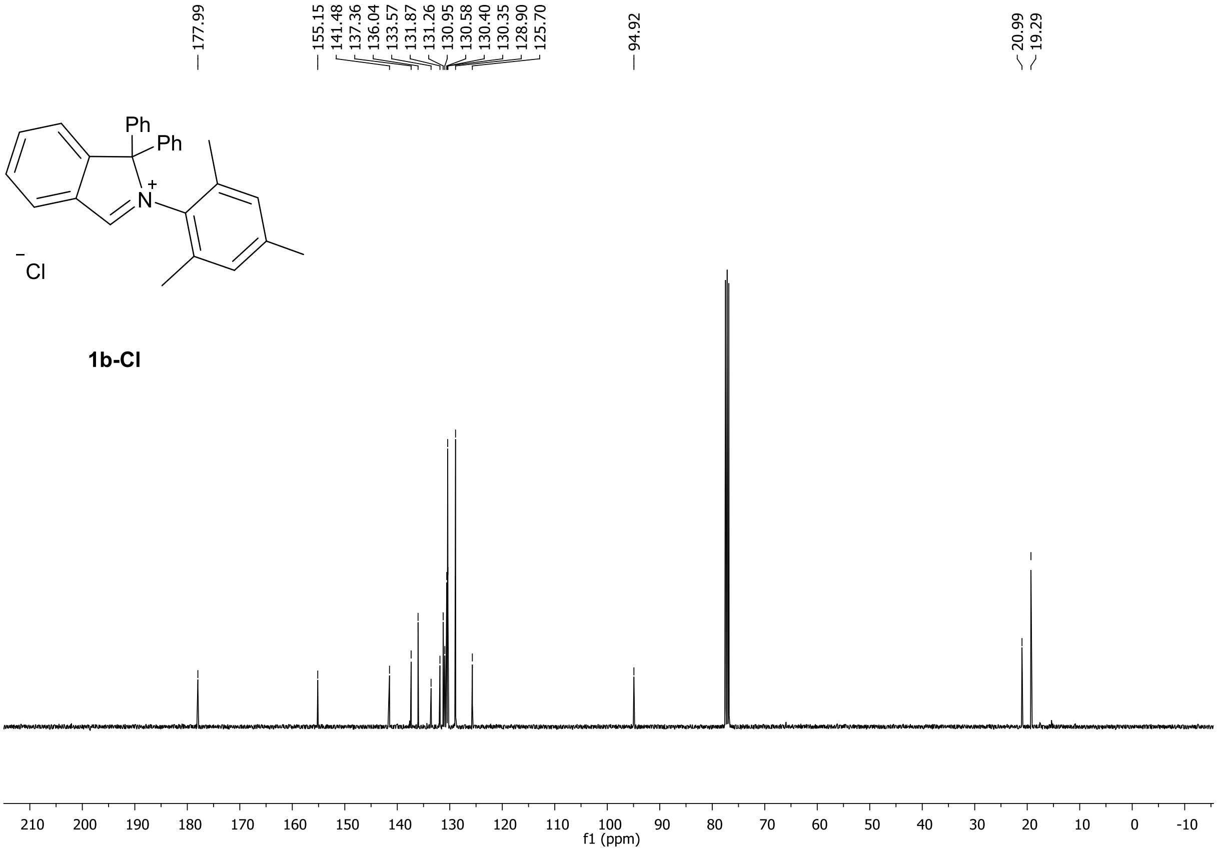

Figure S7. ^{1}H NMR spectra of **2a-OMe** ($CDCl_3$)

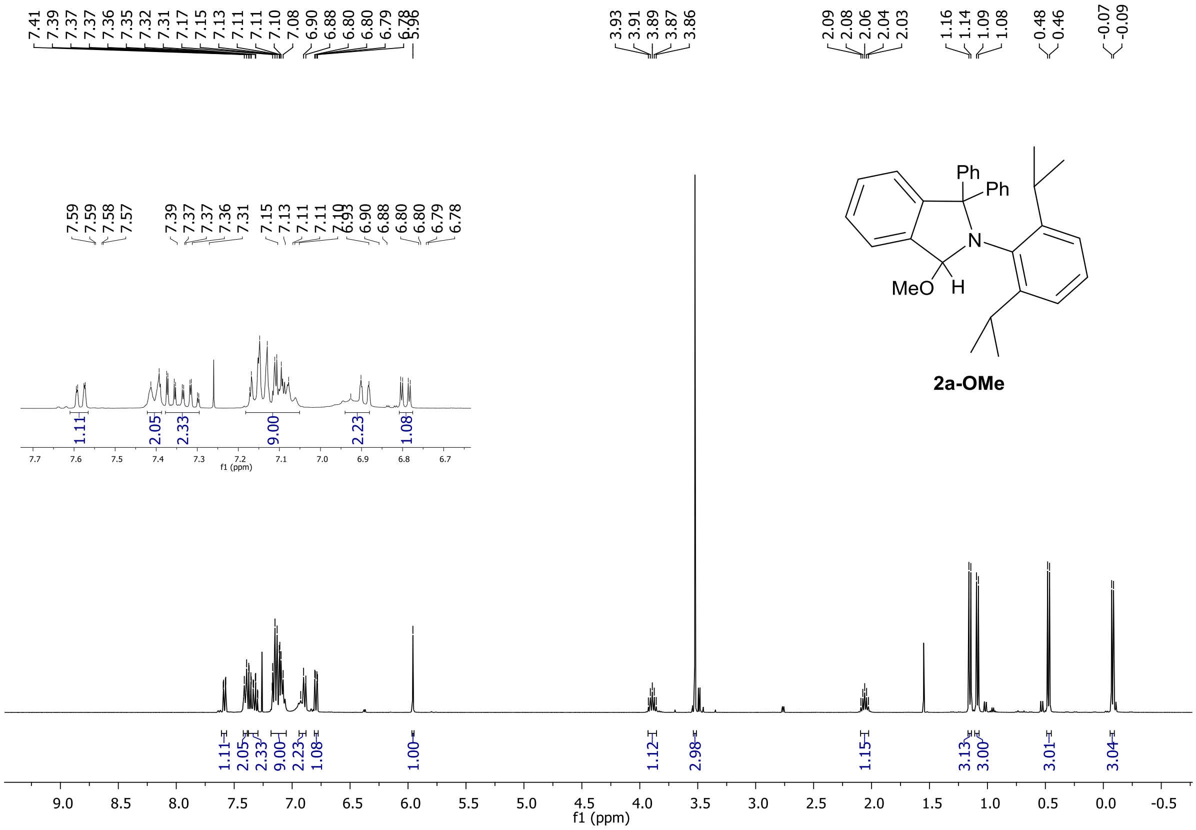

Figure S8. ^{13}C{^{1}H} NMR spectra of **2a-OMe** ($CDCl_3$)

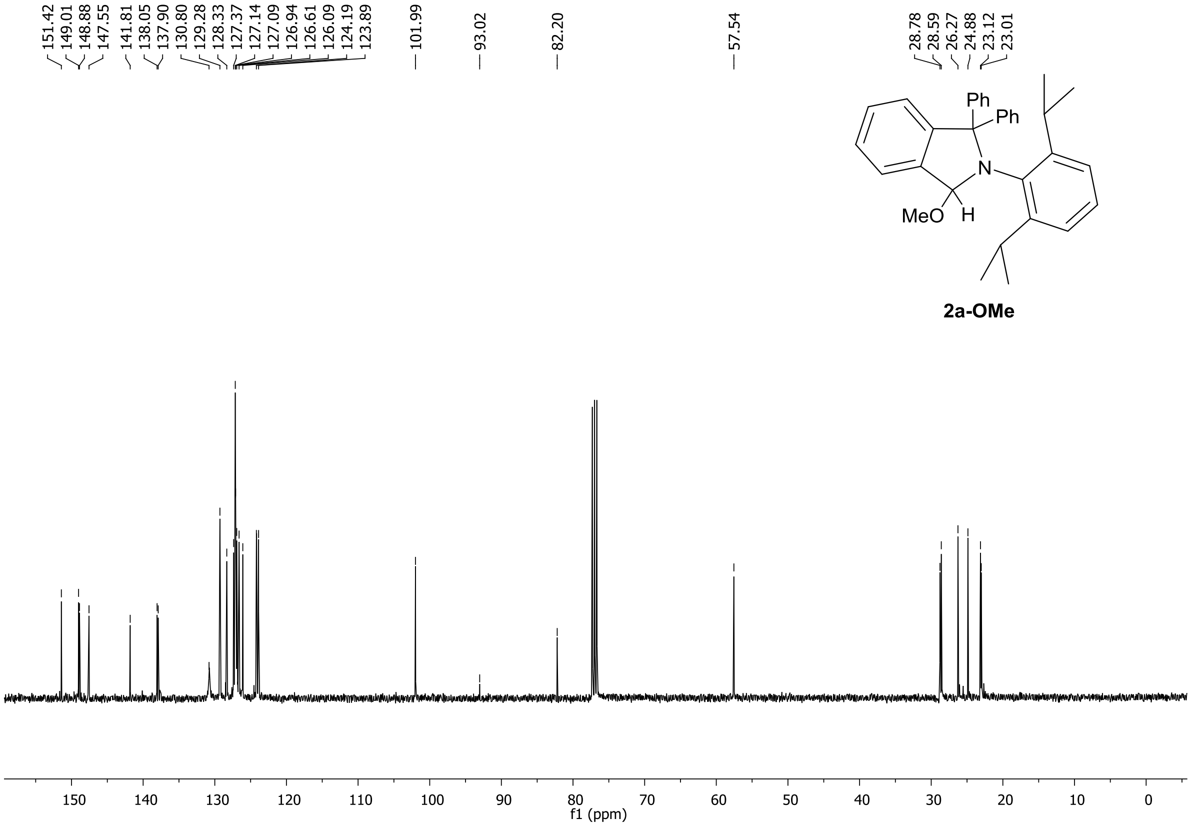

Figure S9. ^{1}H NMR spectra of **2a-OMe** (C_6D_6)

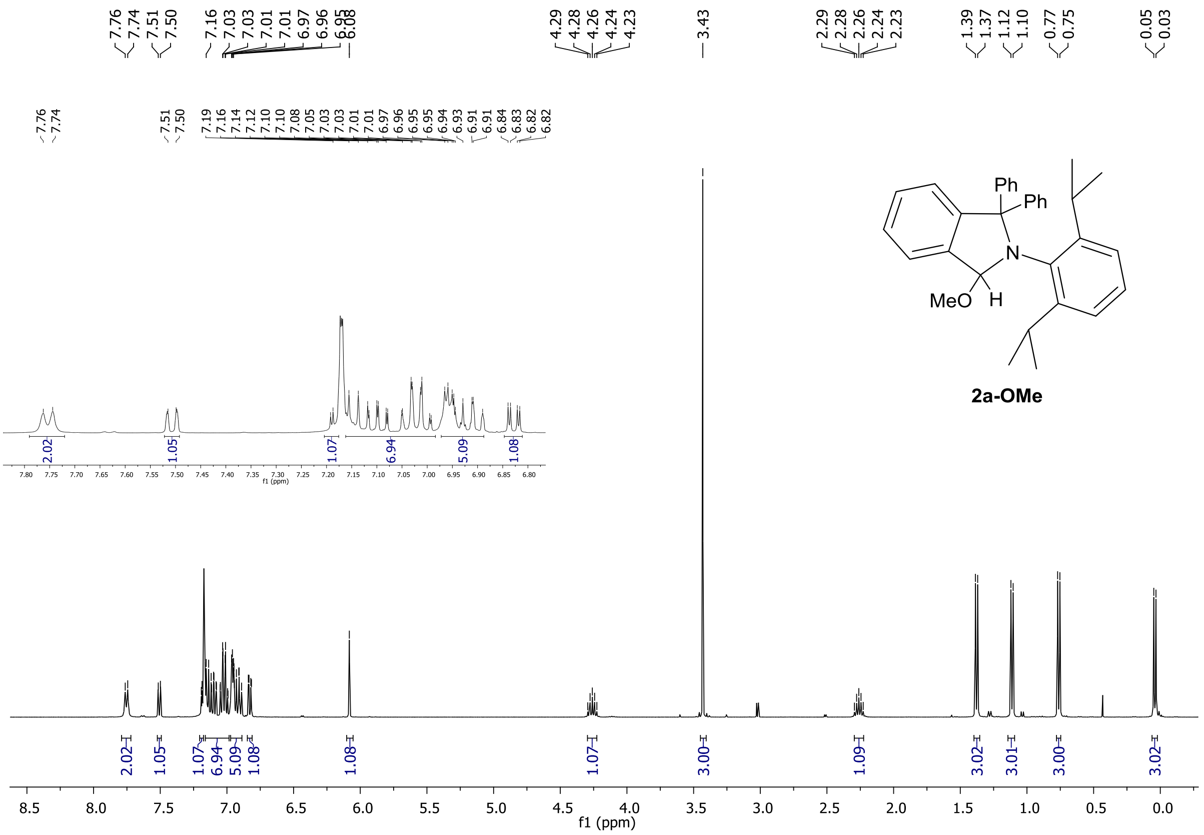

Figure S10. $^{13}C\{^1H\}$ NMR spectra of **2a-OMe** (C_6D_6)

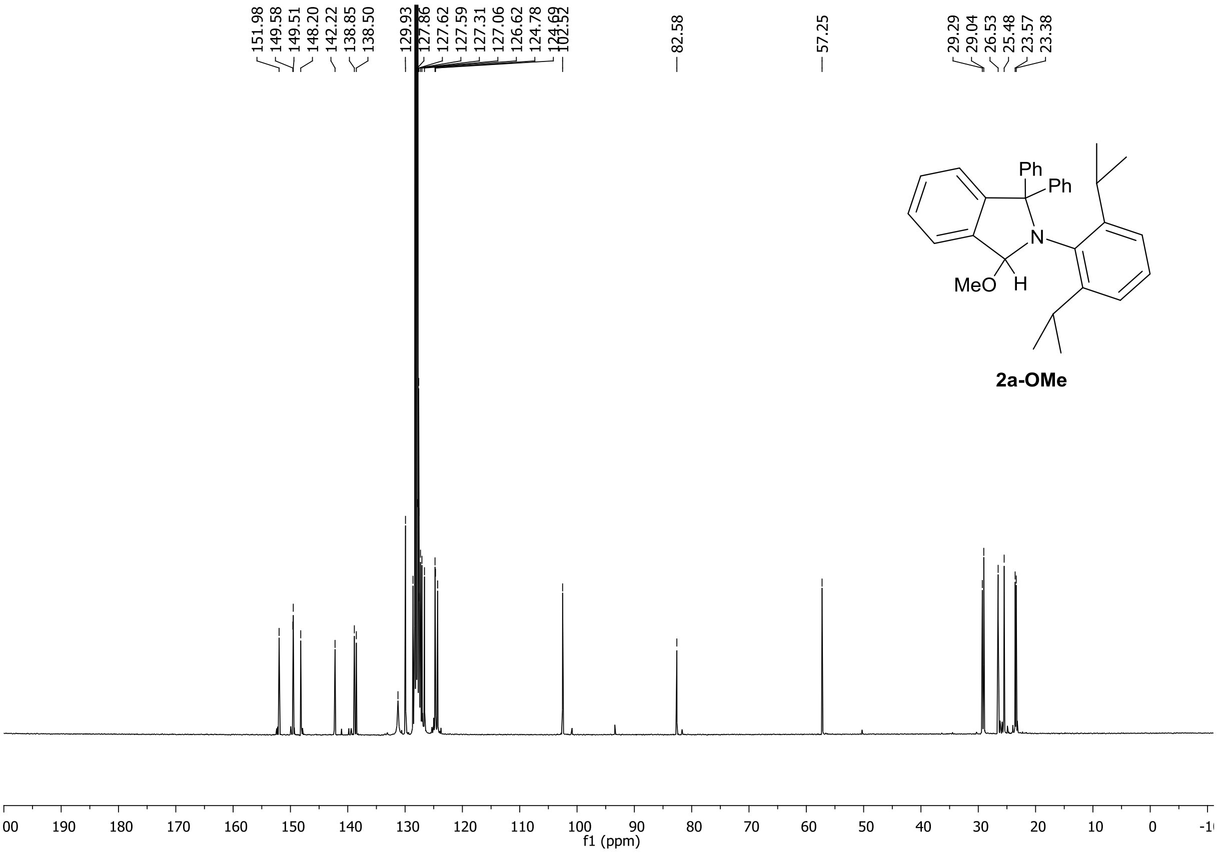

Figure S11. ^{1}H NMR spectra of **2a-OEt** (C_6D_6)

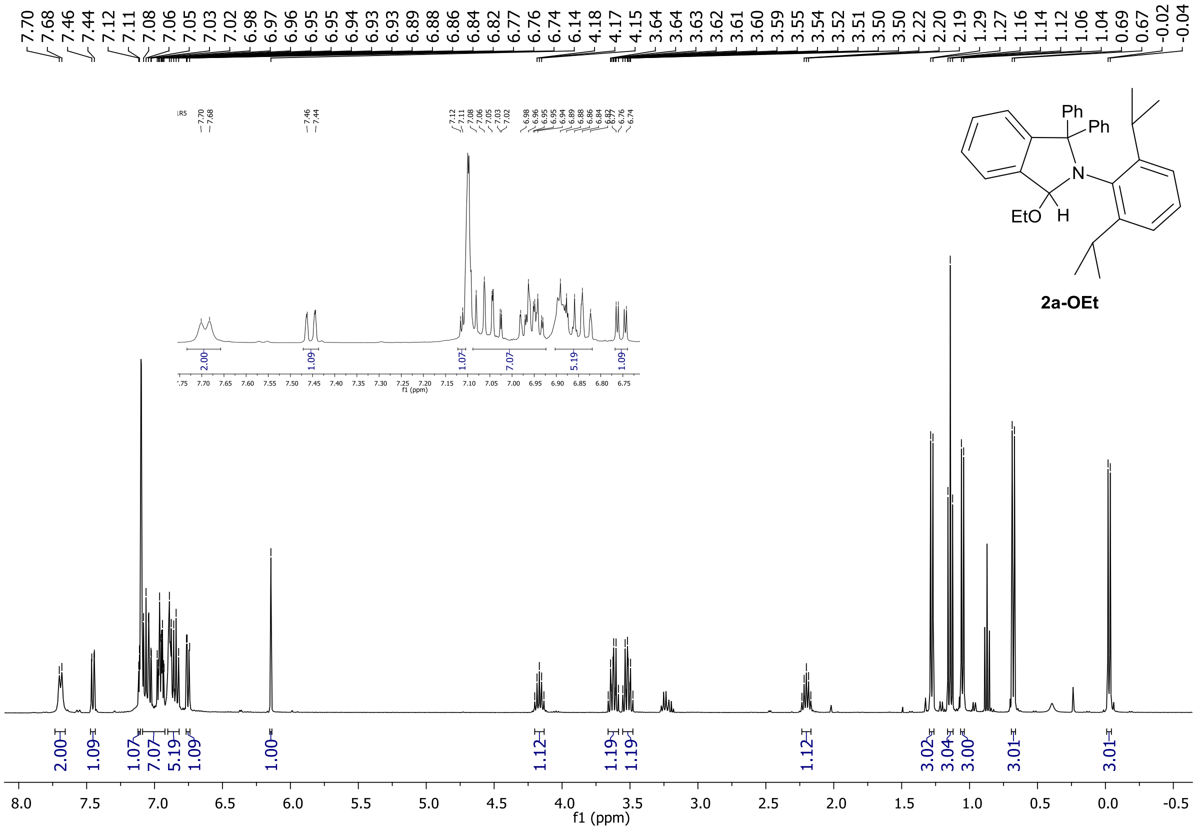

Figure S12. ^{13}C{^{1}H} NMR spectra of **2a-OEt** (C_6D_6)

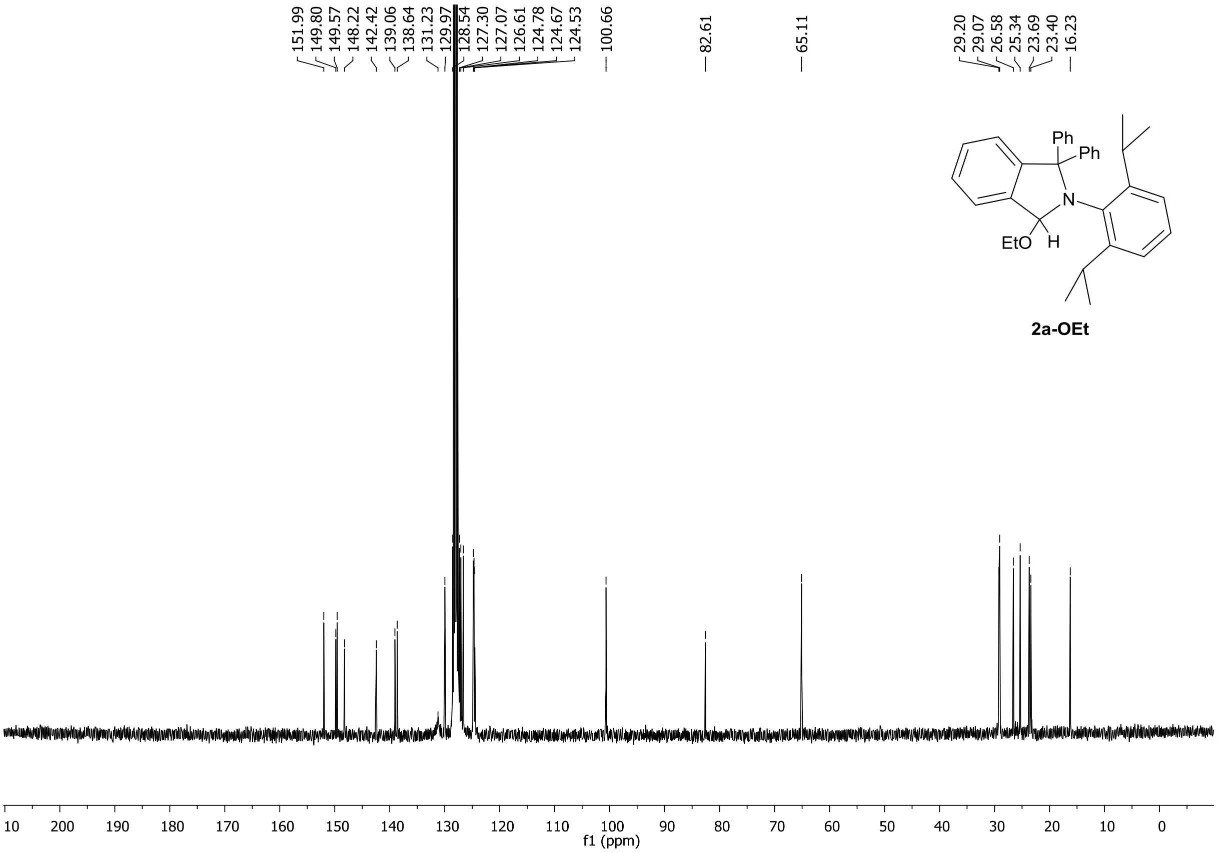

Figure S13. ^{1}H NMR spectra of **2a-O*i*Pr** (C_6D_6)

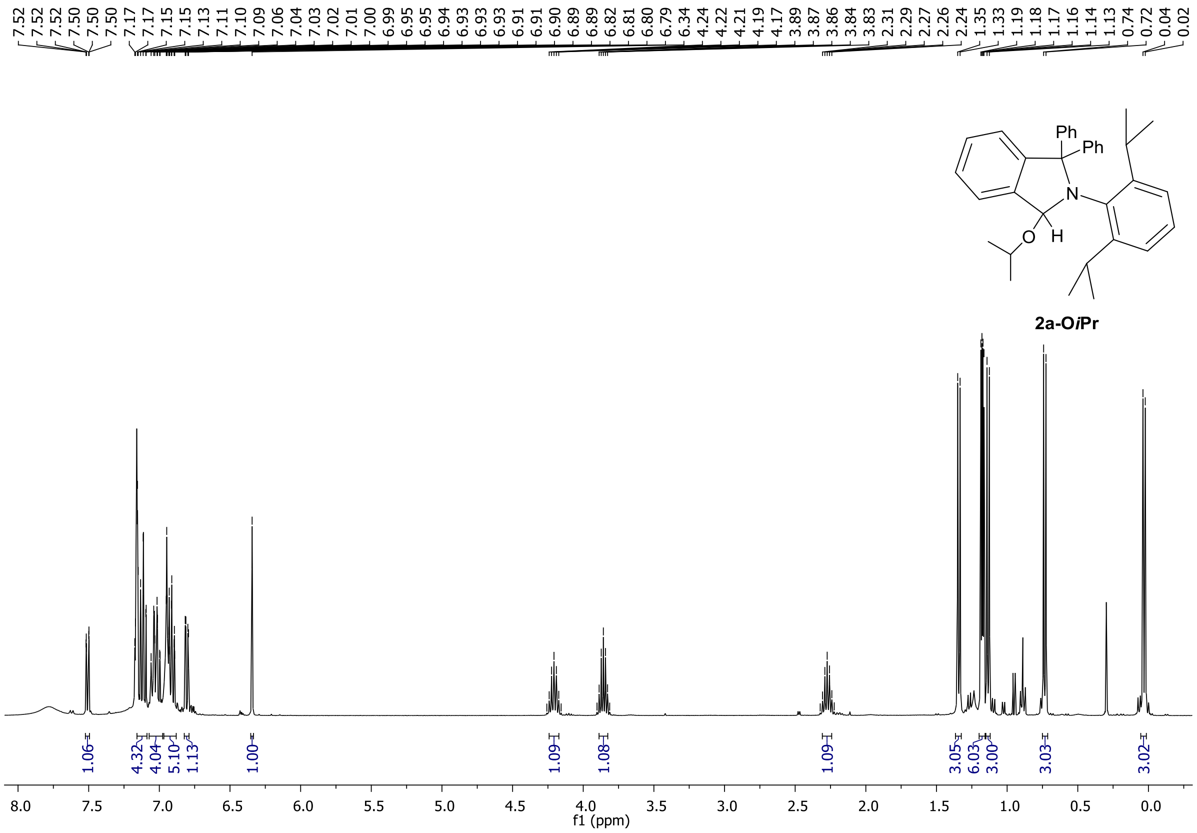

Figure S14. ^{13}C{^{1}H} NMR spectra of **2a-O*i*Pr** (C_6D_6), residue hexane signals are visible

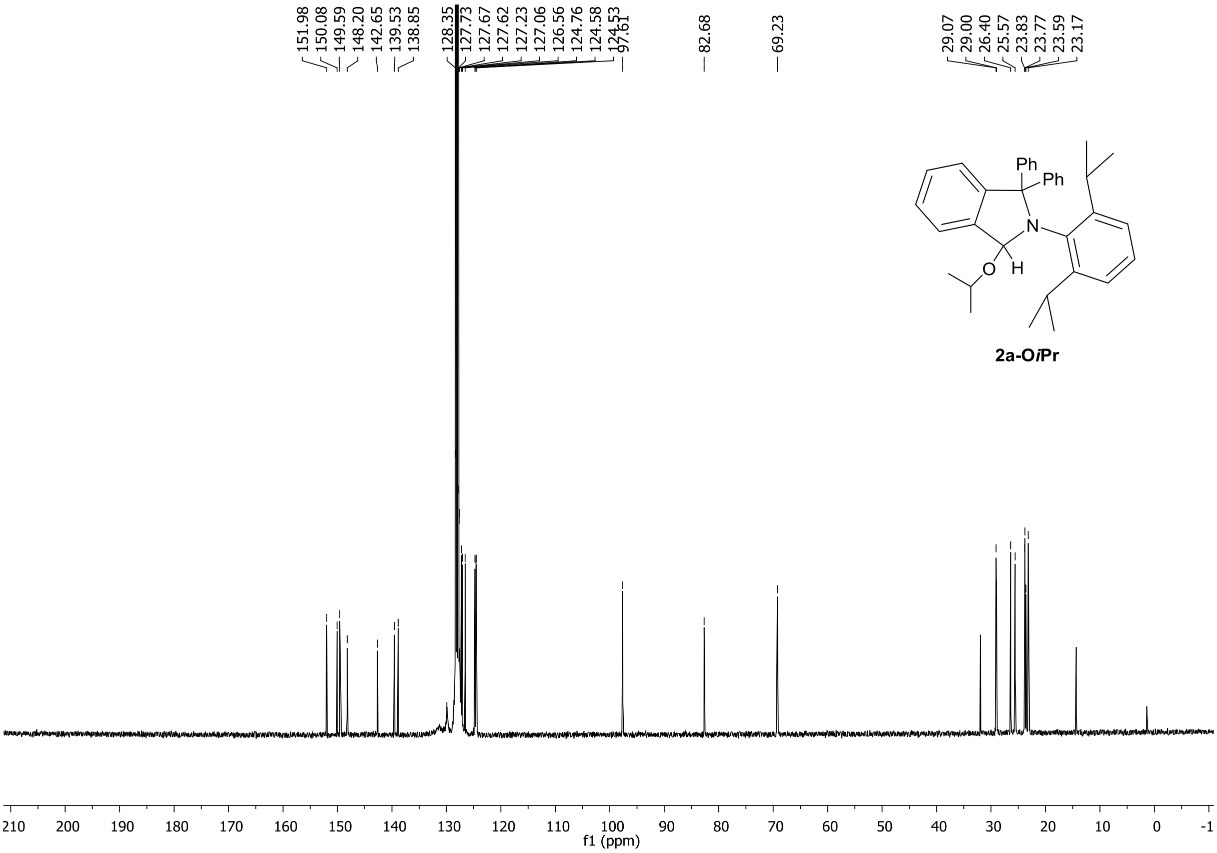

Figure S15. ^{1}H NMR spectra of **2b-OMe** (C_6D_6)

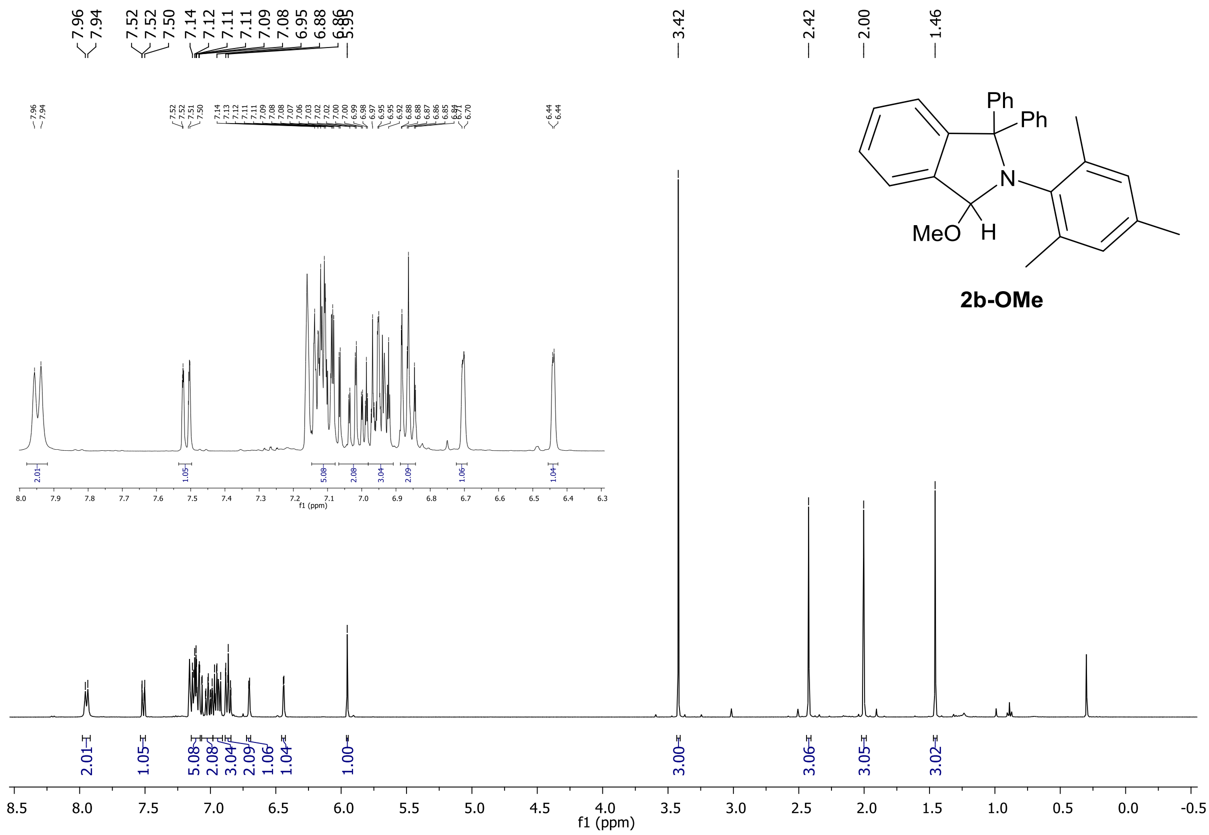

Figure S16. ^{13}C{^{1}H} NMR spectra of **2b-OMe** (C_6D_6)

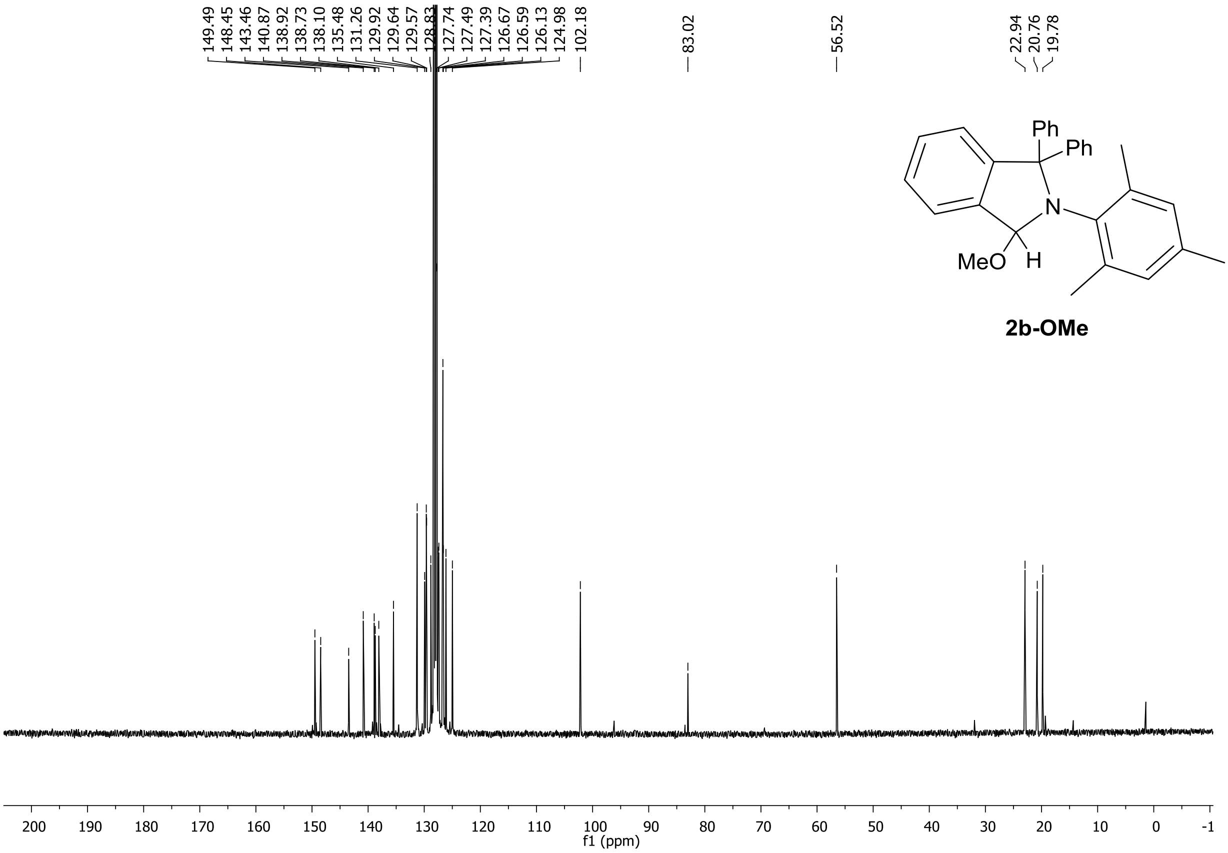

Figure S17. ^{1}H NMR spectra of **2b-OEt** (C_6D_6)

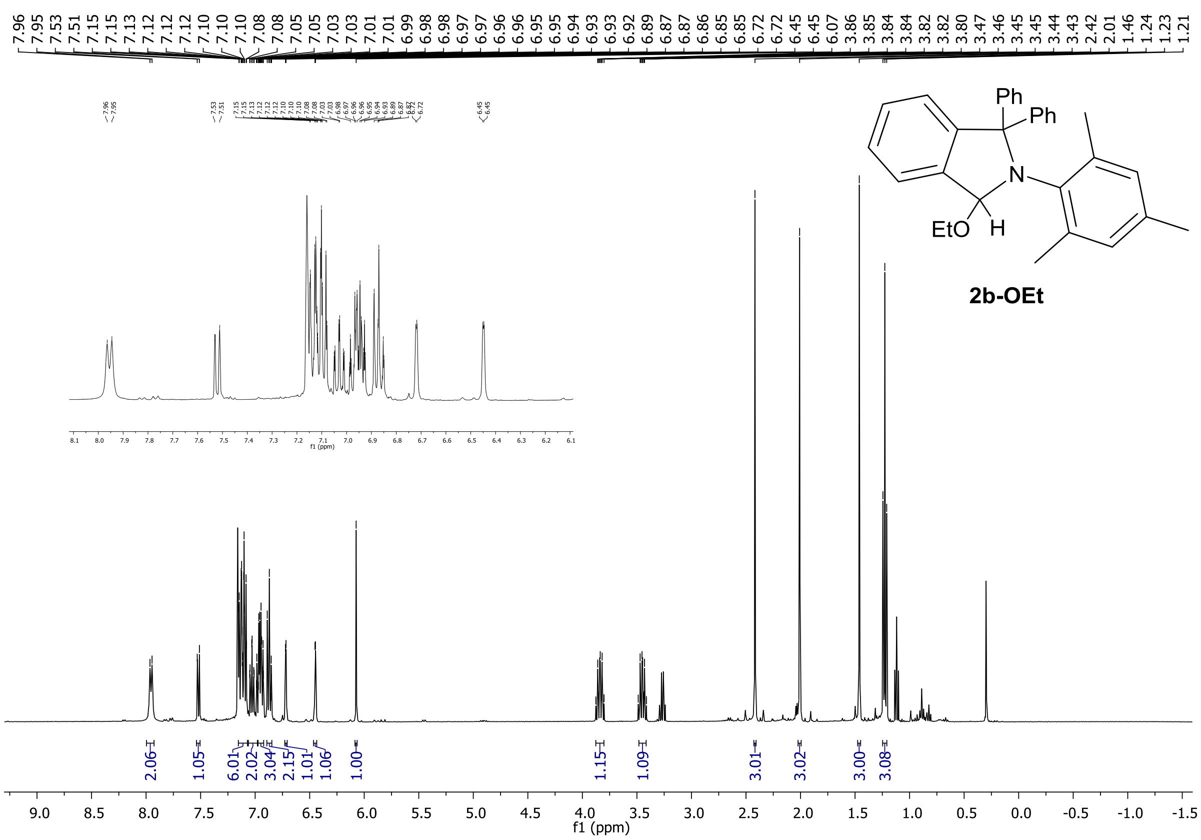

Figure S18. ^{13}C{^{1}H} NMR spectra of **2b-OEt** (C_6D_6)

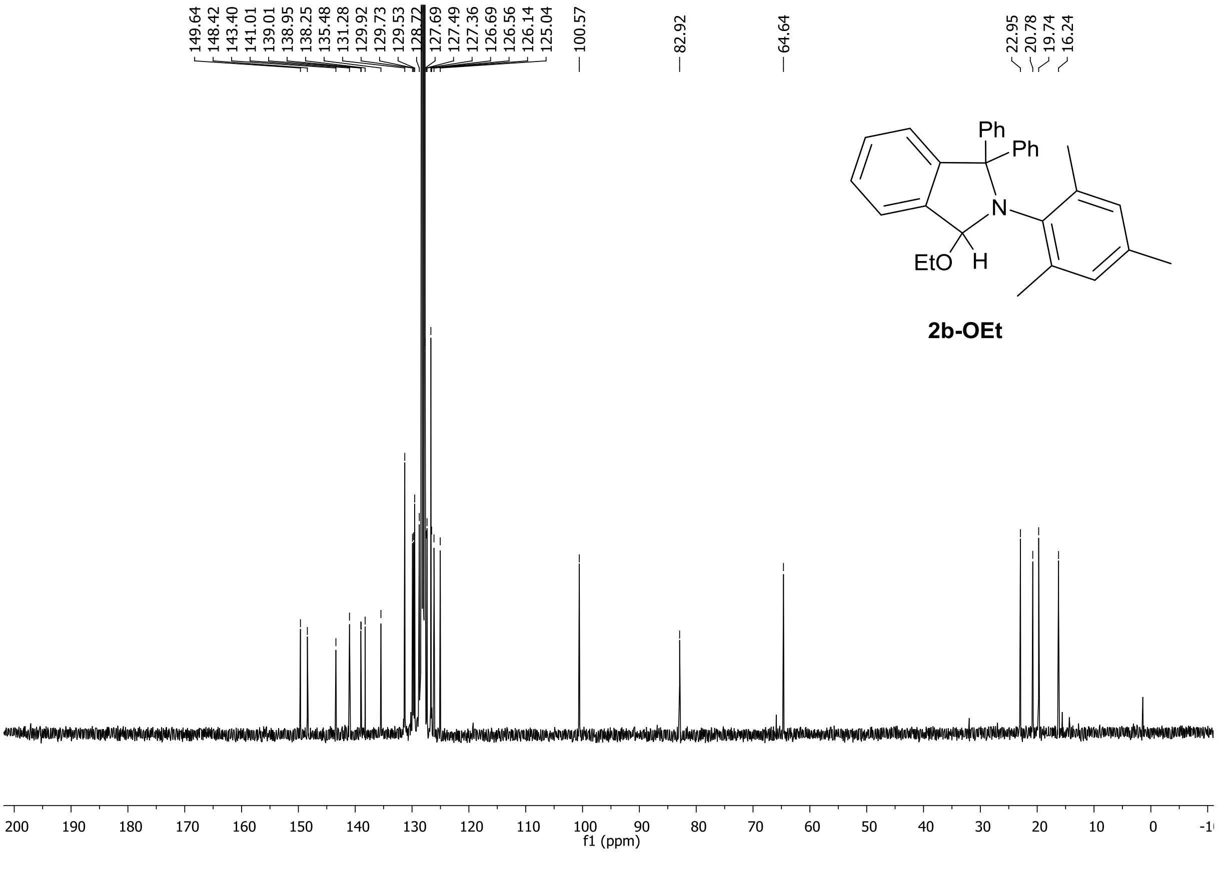

Figure S19. ^{1}H NMR spectra of **2b-O*i*Pr** (CDCl$_3$)

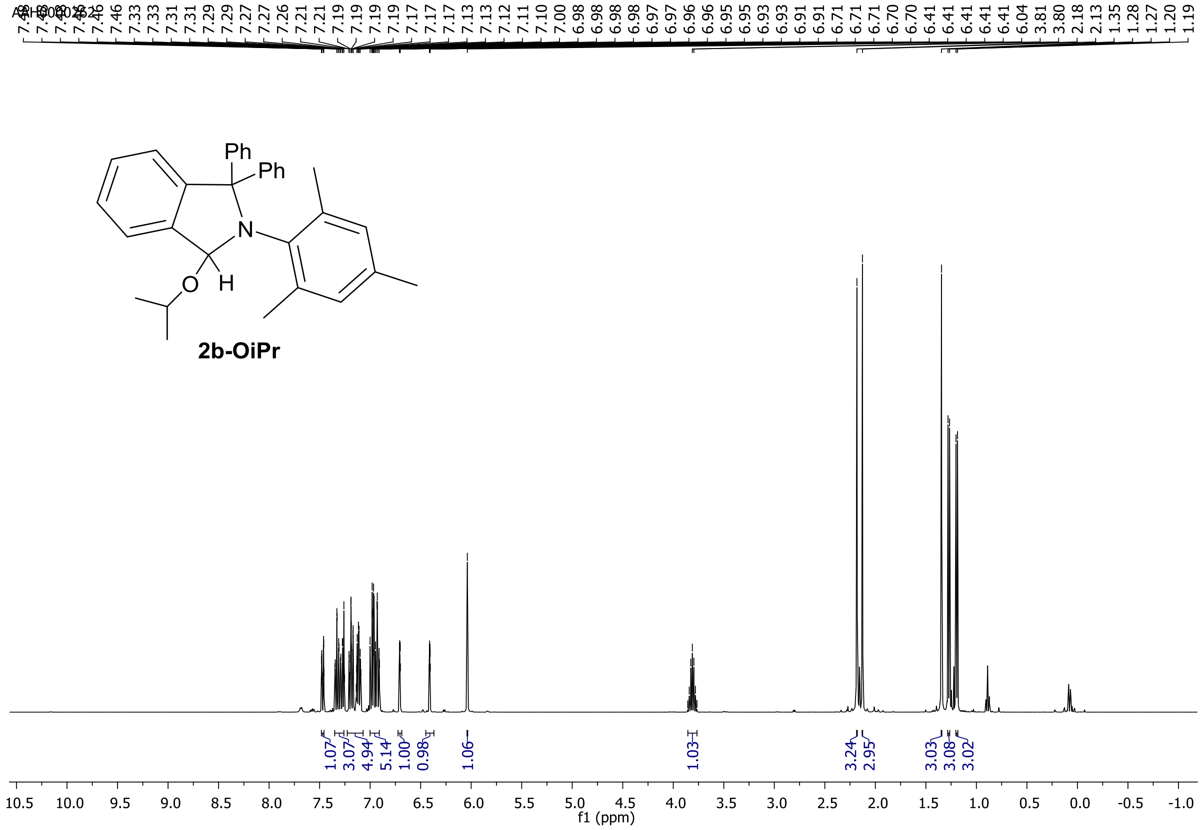

Figure S20. ^{13}C{^{1}H} NMR spectra of **2b-O*i*Pr** (CDCl$_3$)

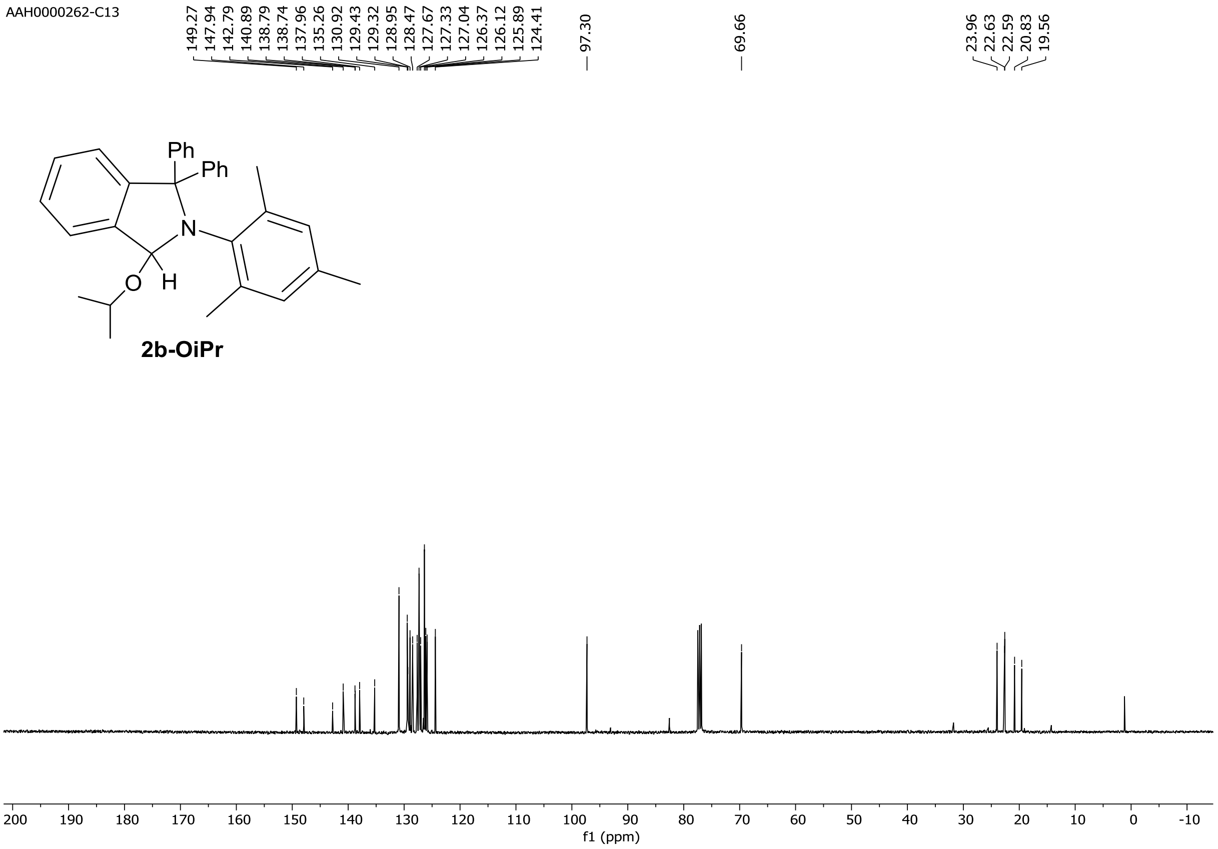

Figure S21. ^{1}H NMR spectra of **3a-S** ($CDCl_3$)

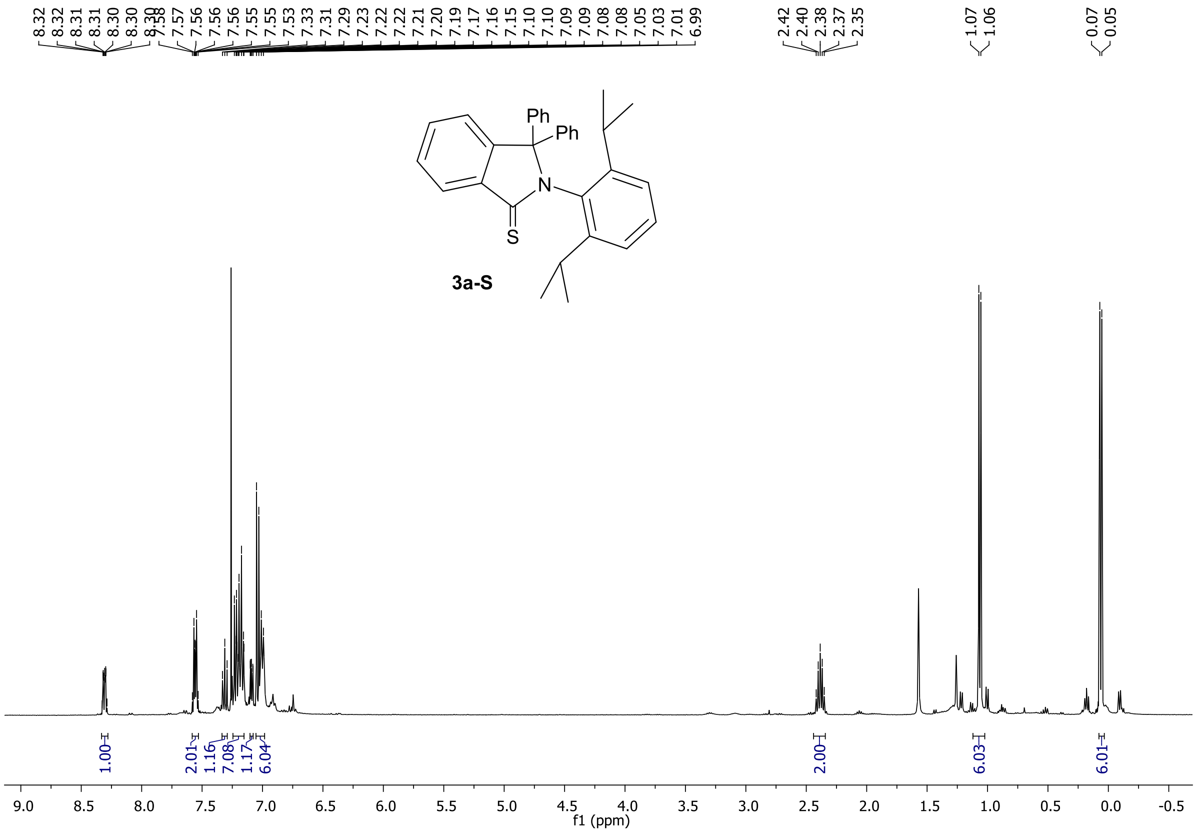

Figure S22. ^{13}C{^{1}H} NMR spectra of **3a-S** ($CDCl_3$)

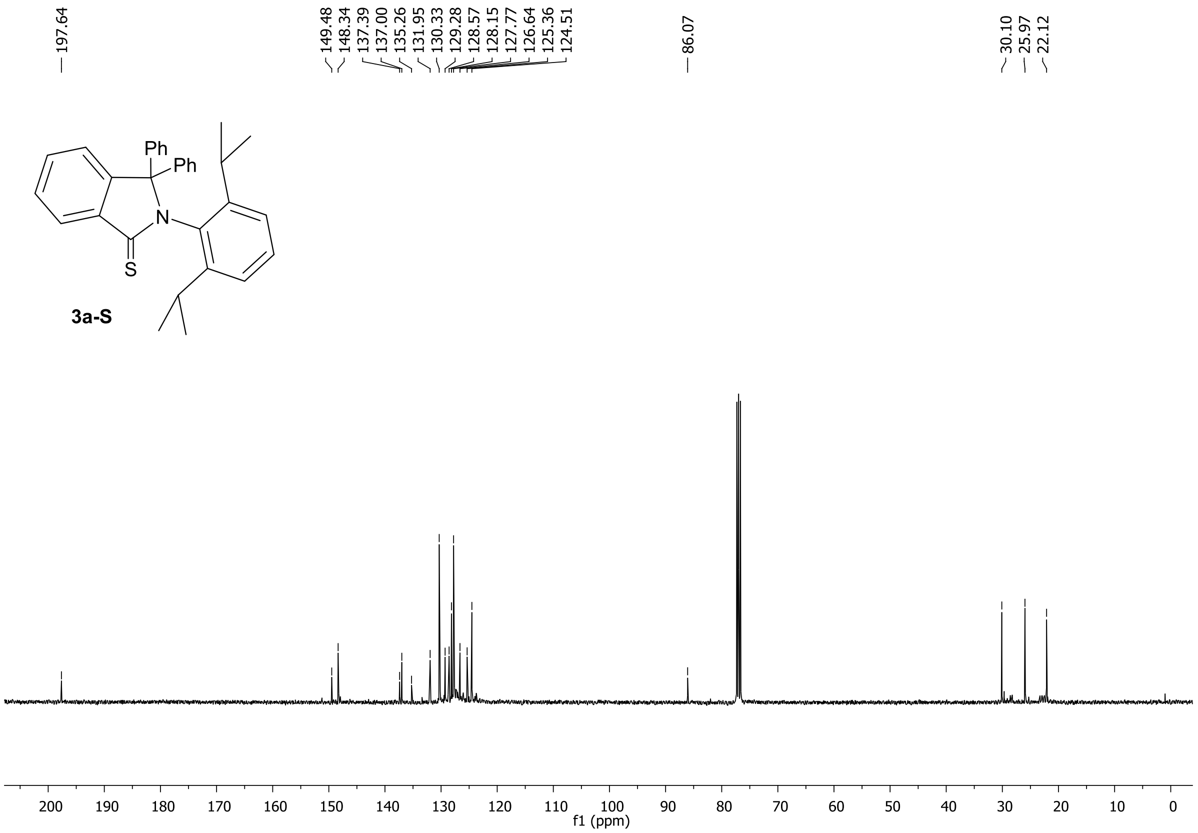

Figure S23. ^{1}H spectra of 4a (C_6D_6)

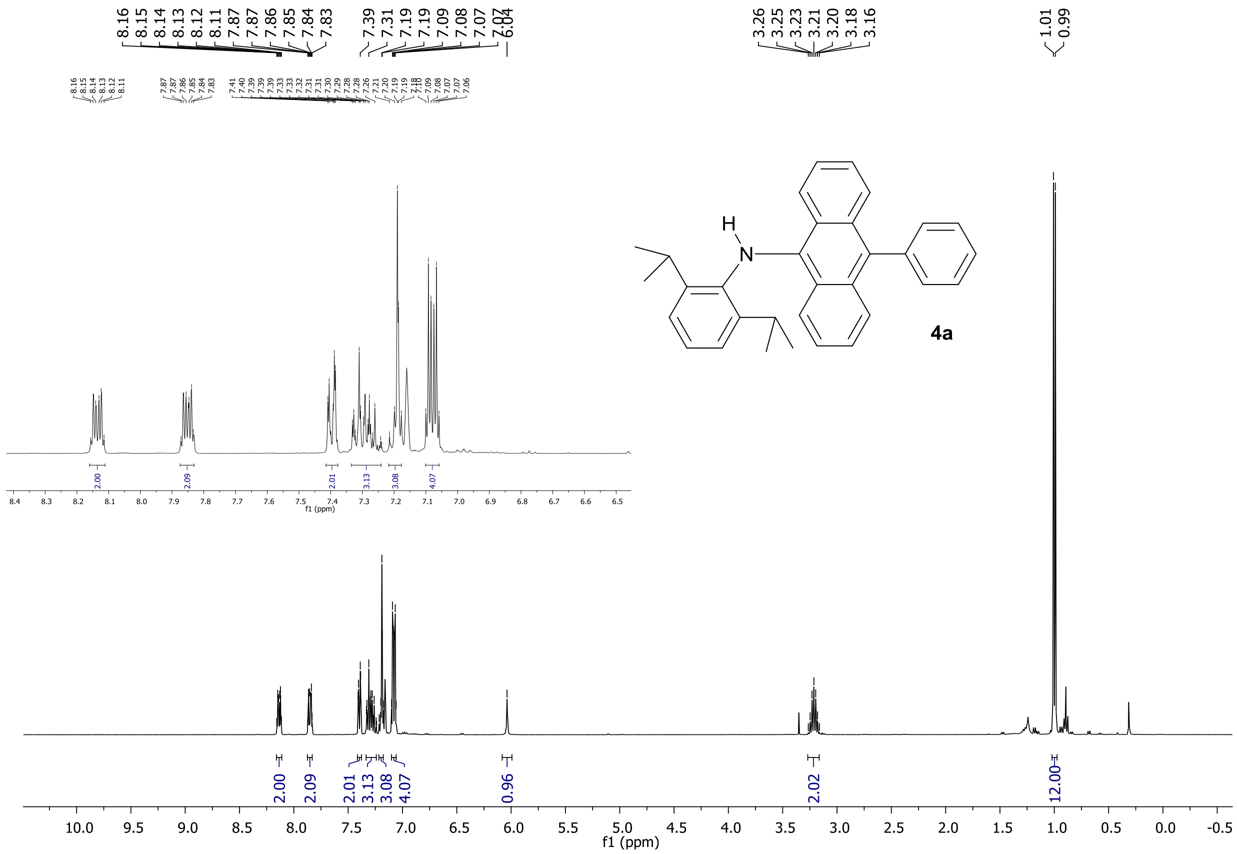

Figure S24. $^{13}C\{^{1}H\}$ NMR spectra of **4a** (C_6D_6), residue hexane signals are visible

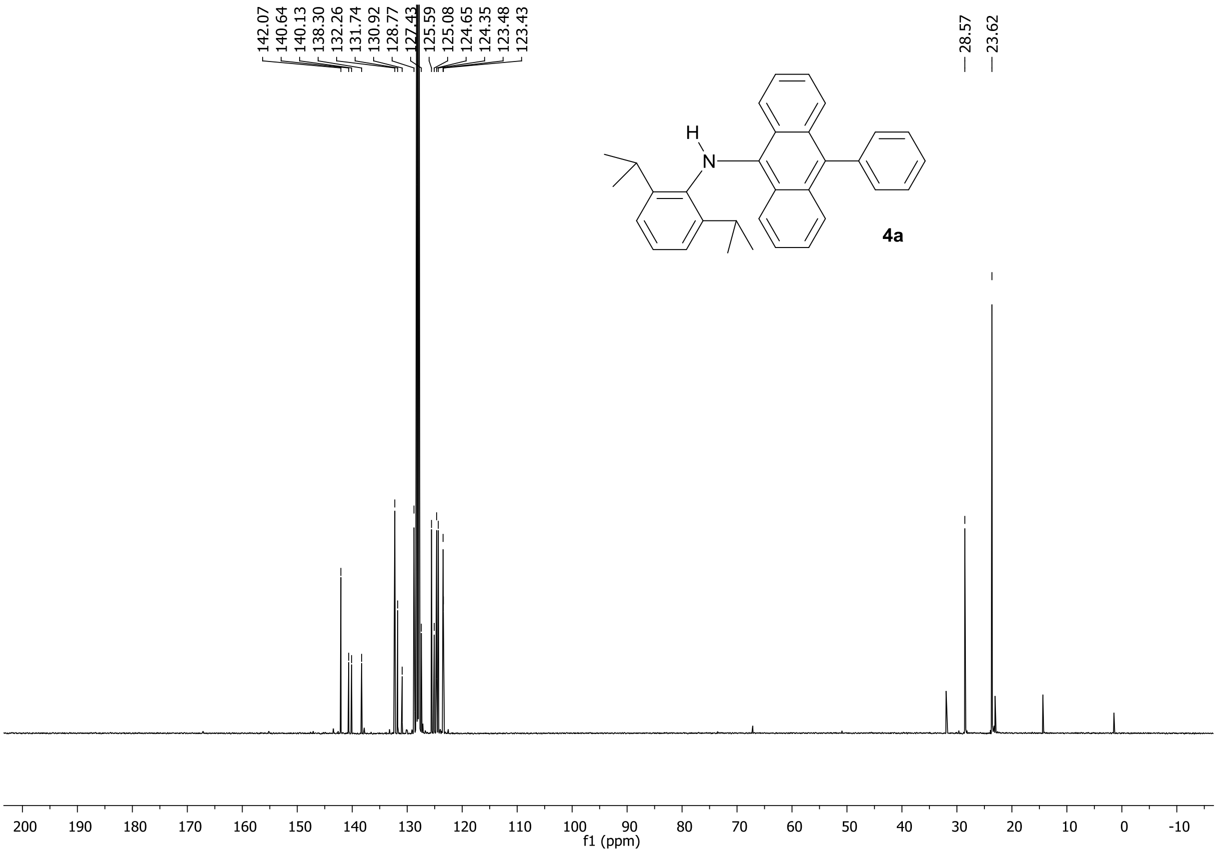

Figure S25. ^{1}H NMR spectra of **4b** (C_6D_6)

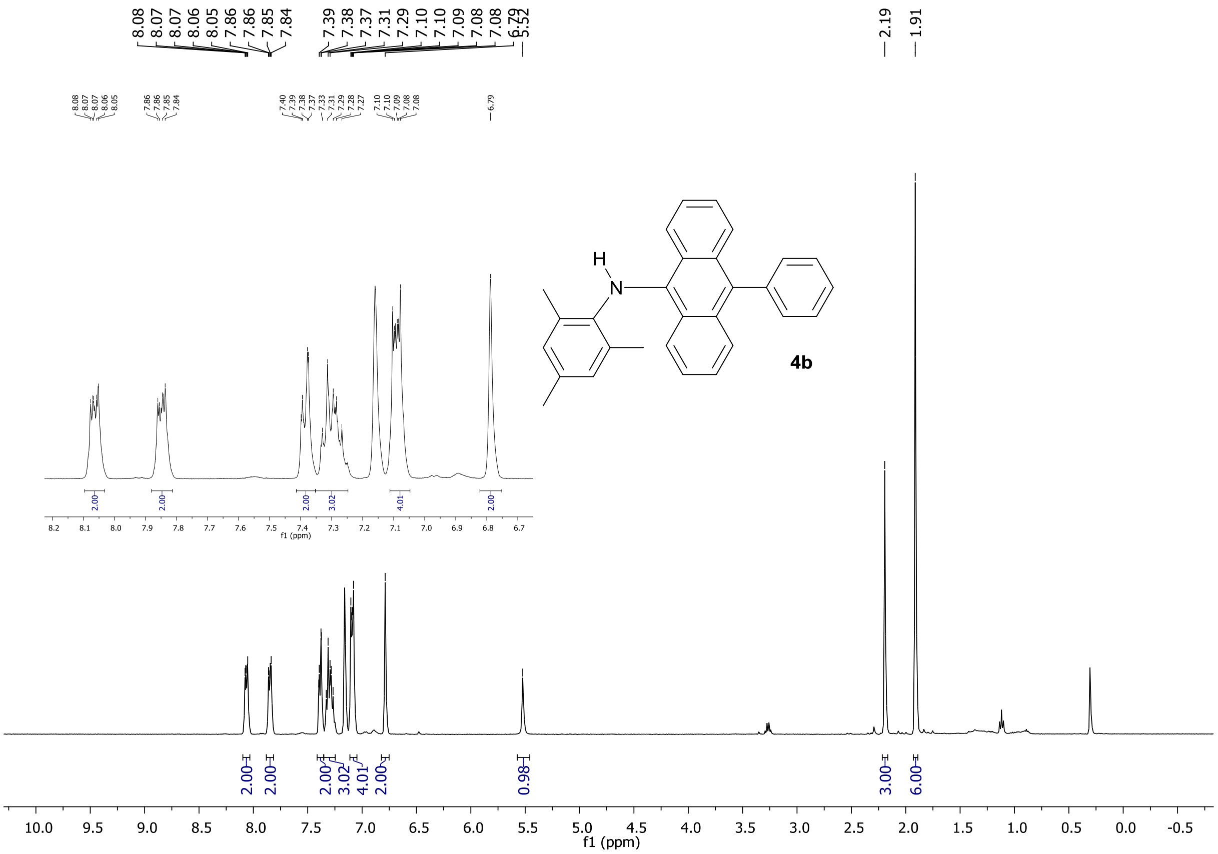

Figure S26. $^{13}C\{^{1}H\}$ NMR spectra of **4b** (C_6D_6)

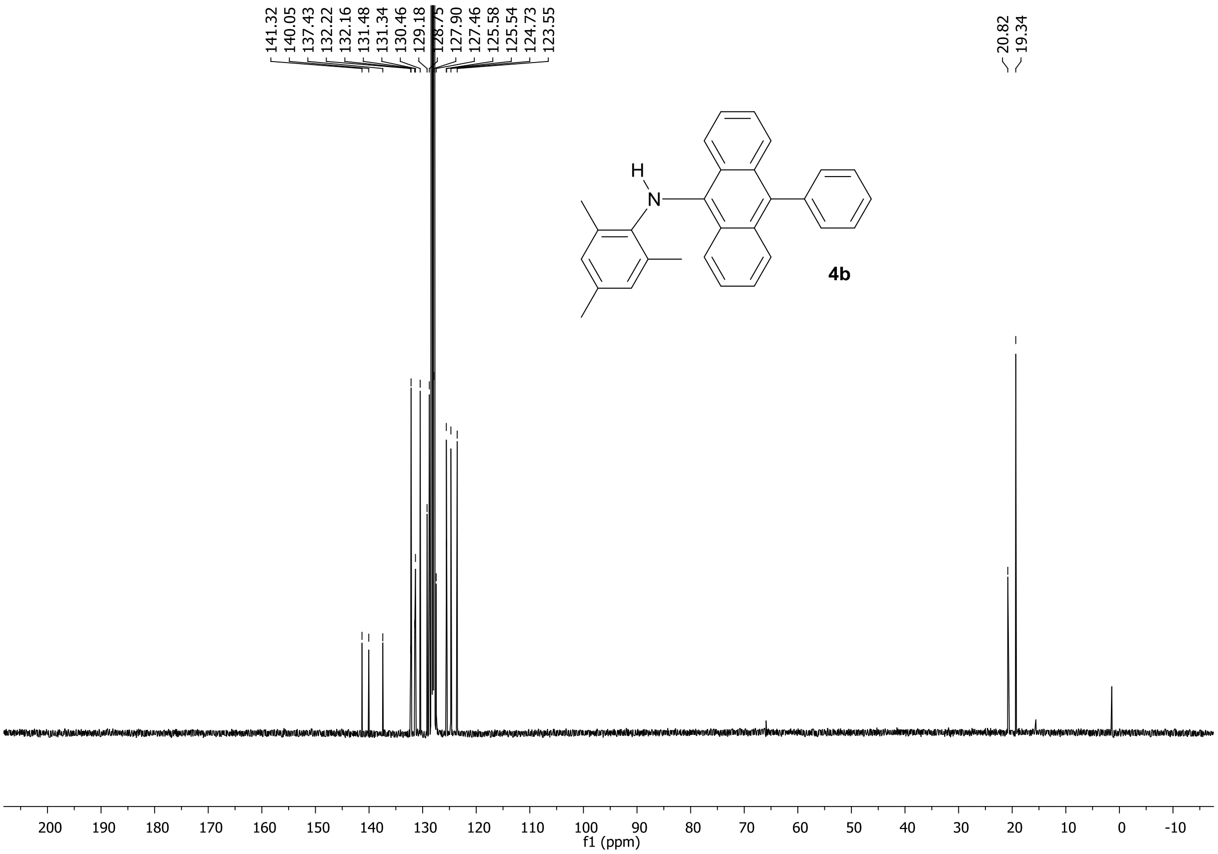

Figure 27. ^{1}H NMR spectra of **5a** ($CDCl_3$)

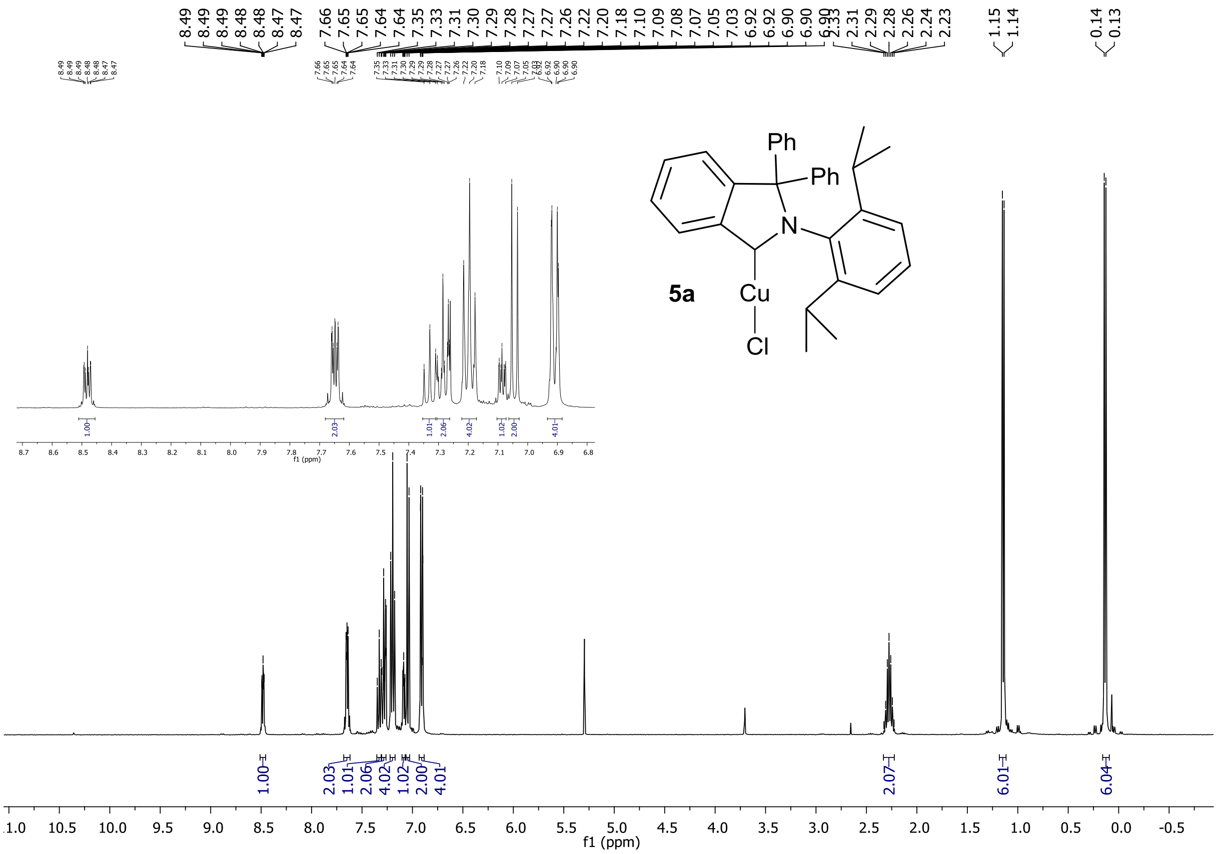

Figure 28. $^{13}C\{^{1}H\}$ NMR spectra of **5a** ($CDCl_3$)

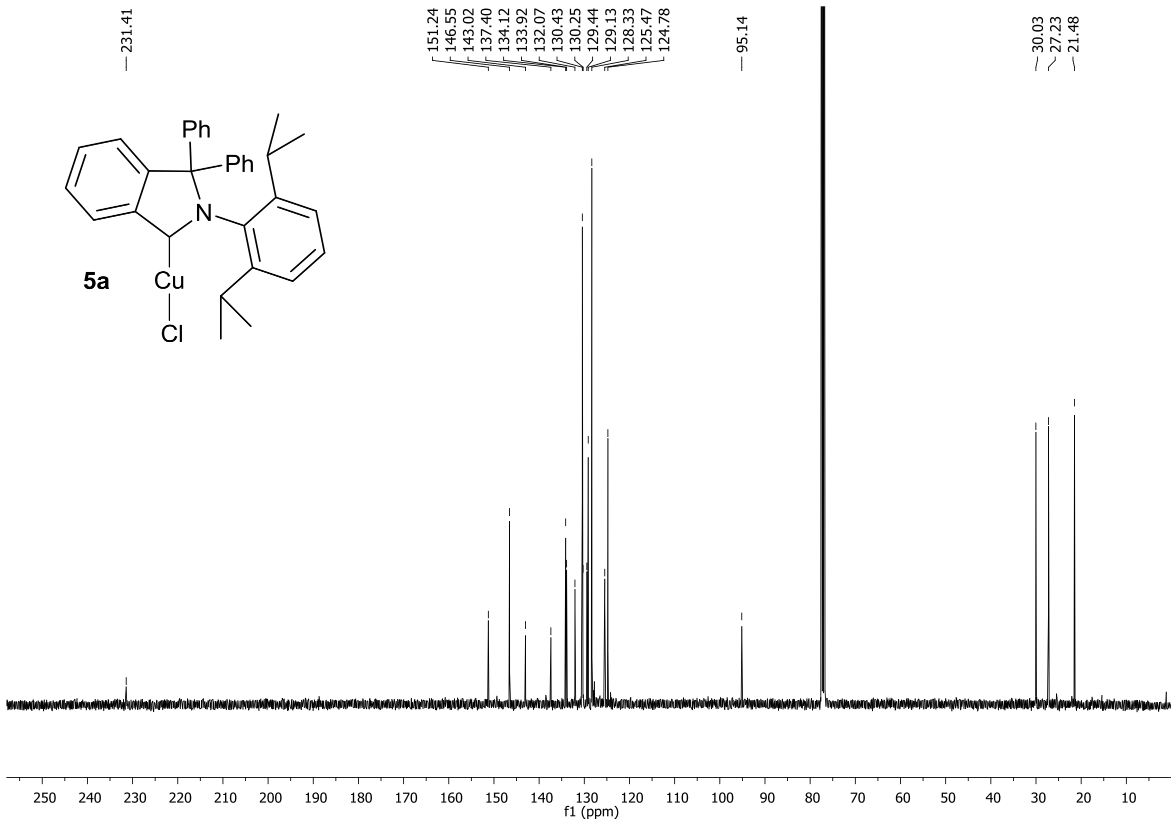

2) Additional experiments and Figures

Thermolysis of 2a-OMe

2a-OMe and anhydrous toluene (5 mL) were placed in a 25 mL Schlenk tube equipped with a magnetic stirring bar and a stopcock and stirred overnight at 130°C. After cooling down to room temperature the solvent was removed under reduced pressure and the residue was taken up with C_6D_6 and analyzed by ^{1}H NMR spectroscopy. According to the NMR spectrum (Figure S27) the residue is a 1:1 mixture of **2a-OMe** and the RER product **4a** .

Figure S29. ^{1}H NMR spectrum of the residue obtained from the thermolysis of **2a-OMe**

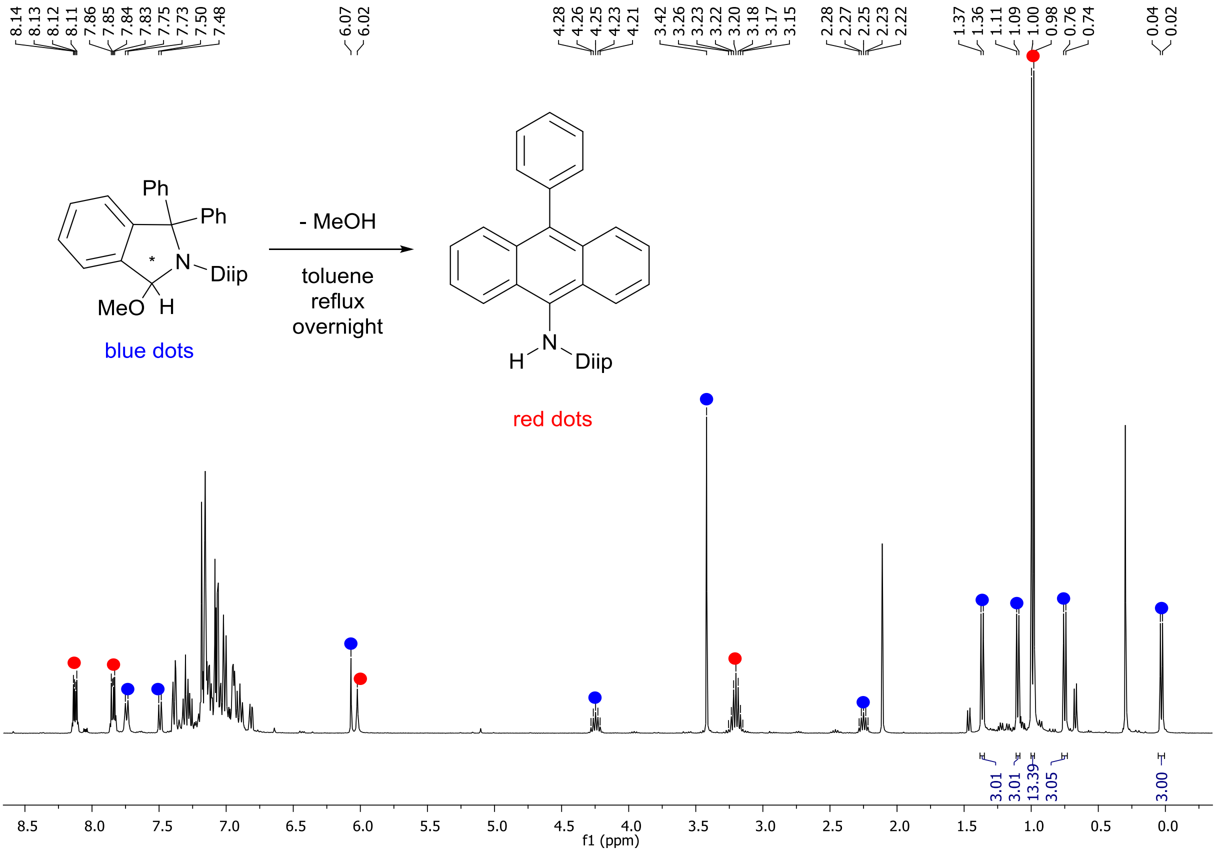

Reaction of 1a-Cl with CuOAc

In a 25 mL Schlenk tube equipped with a magnetic stirring bar and a stopcock 50 mg (XXX mmol) of **1a-Cl,** 20 mg of CuOAc (XX mmol; 1.5 equiv.) and anhydrous dioxane (5 mL) were added. The reaction mixture was stirred overnight at 120°C, cooled down to room temperature and filtered through a pad of Celite. The solvent of the filtrate was removed under reduced pressure and the residue was taken up with C_6D_6 and analyzed using ^{1}H NMR spectroscopy. The residue is a 2:1 mixture of **5a** and RER product **4a** according to the ^{1}H NMR spectrum (Figure S28).

Figure S30. ^{1}H NMR spectrum of the residue obtained from the reaction of **1-aCl** with CuOAc.

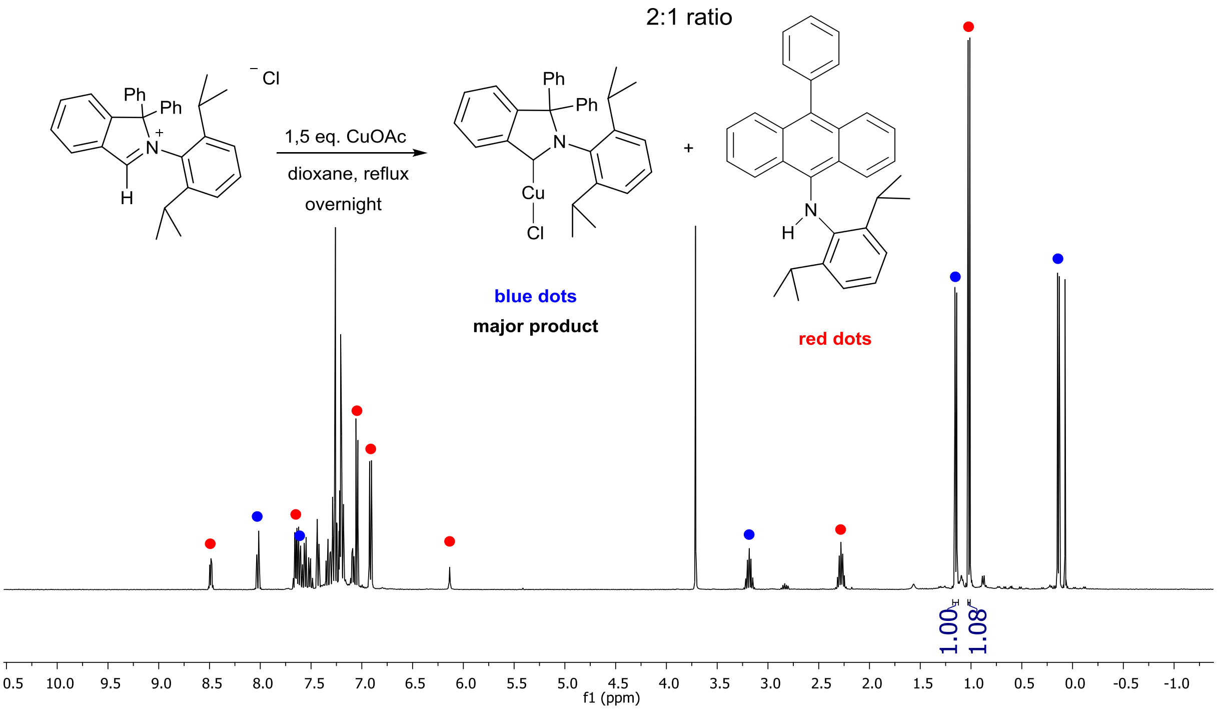

Reaction of 1a-Cl with LiHMDS

A 25 mL Schlenk tube equipped with a magnetic stirring bar and a stopcock was charged with 100 mg (XXX mmol) of **1a-Cl** and 36 mg (XXX mmol, 1 eqiv.) LiHMDS. Afterwards the flask was cooled down to -78°C and 5ml of precooled THF was added. Then the Schlenk tube was removed from the cooling bath and the reaction mixture was stirred overnight at RT. The solvent was removed under reduced pressure and the residue was taken up with toluene and subsequently filtered through a pad of Celite. Solvent evaporation of the filtrate under reduced pressure afforded crude **4a** in approximately 92% (see Figure S29).

Figure S31. ^{1}H-NMR spectra of **4a** received from **1-aCl** and LiHMDS

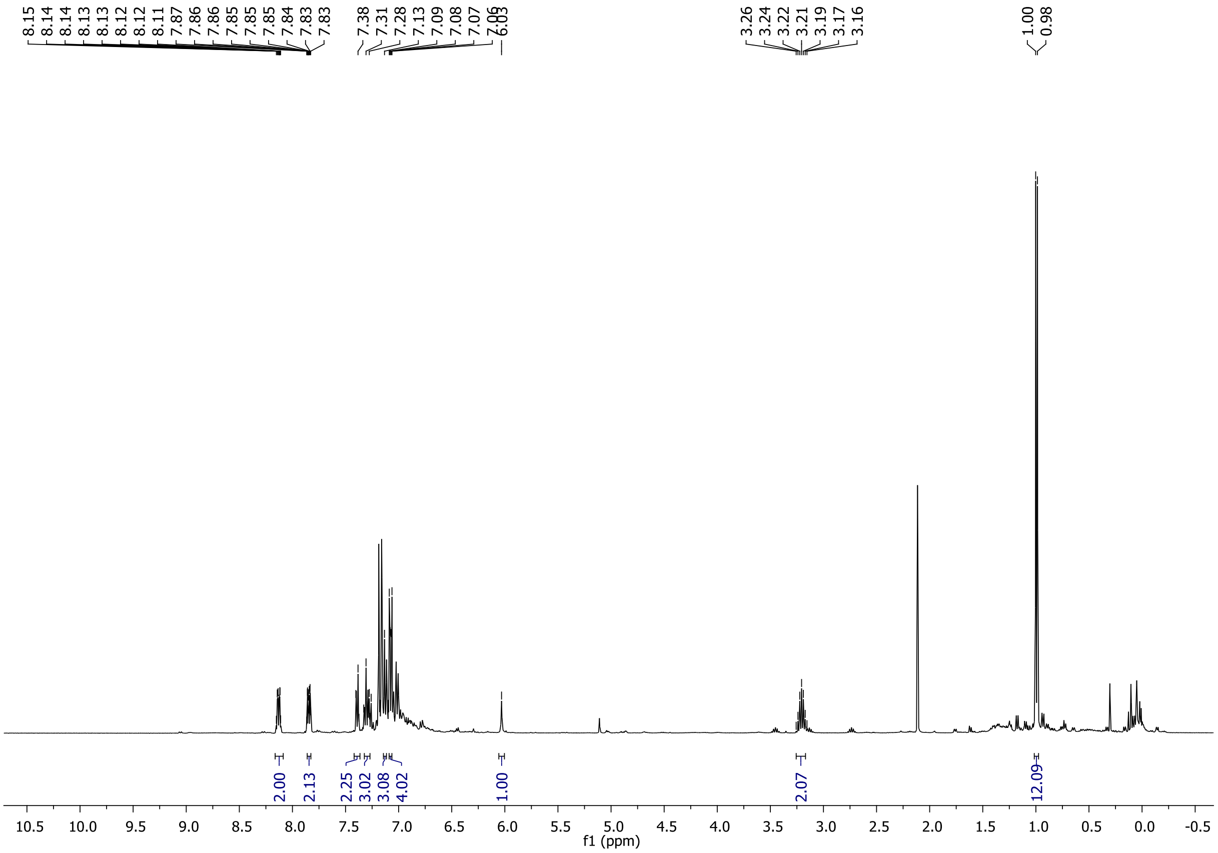

Figure S32. Comparison of the iminium proton shifts at ^{1}H NMR of **1a-OTf** and **1a-Cl** in $CDCl_3$

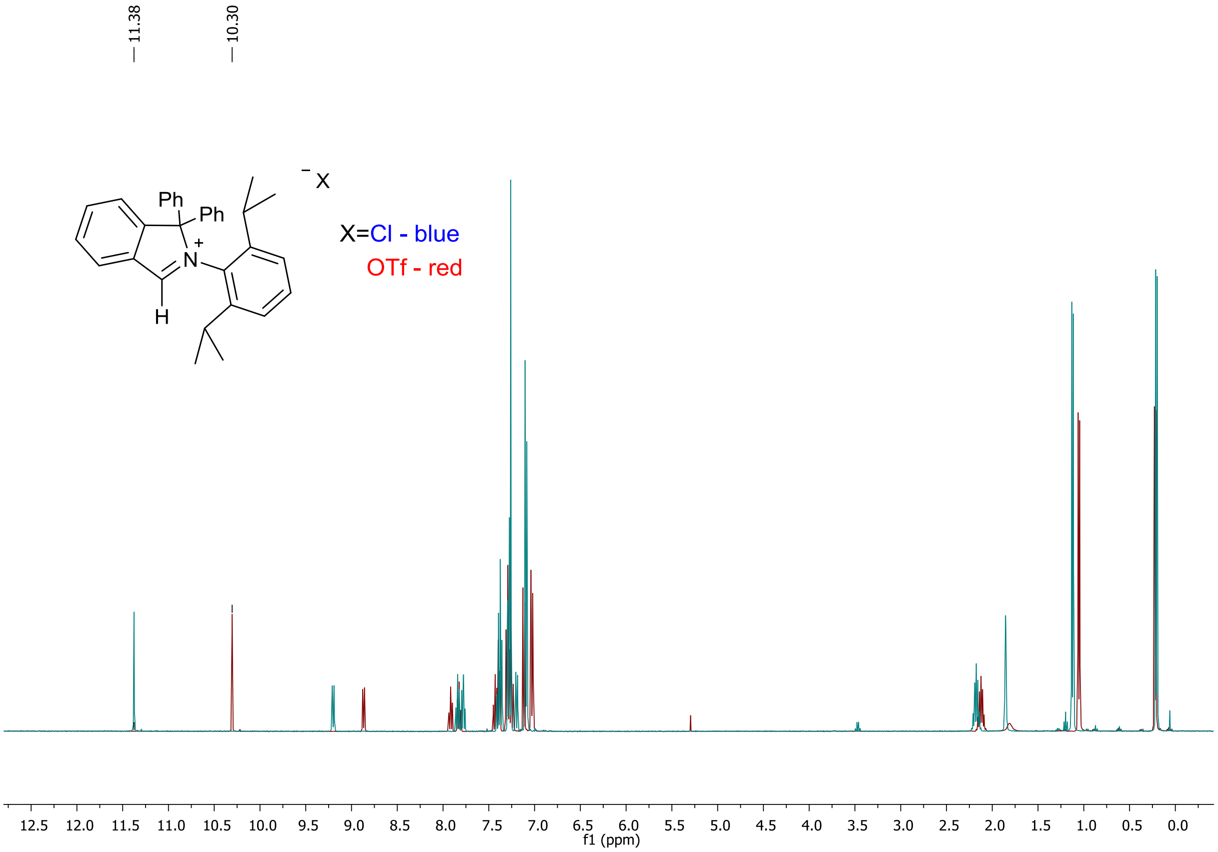

3) X-ray crystallography

Diffraction data were collected by the ω-scan technique, for **2a-O*i*Pr** and **1a-Cl** at 100(1) K, for **2a-OMe** at room temperature on Rigaku Xcalibur four-circle diffractometer with Eos CCD detector and graphite-monochromated MoK_{α} radiation (λ=0.71069 Å), and for **1a-OMe** and **1a-OEt** at room temperature on Rigaku SuperNova four-circle diffractometer with Atlas CCD detector and mirror-monochromated CuK_{α} radiation (λ=1.54178 Å). The data were corrected for Lorentz-polarization as well as for absorption effects [S2]. Precise unit-cell parameters were determined by a least-squares fit of reflections of the highest intensity, chosen from the whole experiment. The structures were solved with SHELXT [S3] and refined with the full-matrix least-squares procedure on F^2 by SHELXL-2013 [S4]. All non-hydrogen atoms were refined anisotropically, all hydrogen atoms were placed in idealized positions and refined as 'riding model' with isotropic displacement parameters set at 1.2 (1.5 for methyl groups) times U_{eq} of appropriate carrier atoms. Crystallographic data have been deposited with the Cambridge Crystallographic Data Centre, Nos. CCDC 1857164 (**2a-OiPr**) 1857165 (**1a-Cl**) 1857166 (**2a-OMe**), 1857167 (**2a-OEt**), 1857168 (**2b-OMe**), 1921589 (**4b**), and 1921590 (**5a**). Copies of this information may be obtained free of charge from: The Director, CCDC, 12 Union Road, Cambridge, CB2 1EZ, UK. Fax: +44(1223)336-033, e-mail:deposit@ccdc.cam.ac.uk, or www: www.ccdc.cam.ac.uk.

Table S1. Crystal data, data collection and structure refinement

Compound	**1a-Cl**	**2a-OMe**	**2a-OEt**	**2a-O*i*Pr**	**2b-OMe**	**4b**	**5a**
Formula	$C_{32}H_{32}ClN$	$C_{33}H_{35}NO$	$C_{34}H_{37}NO$	$C_{35}H_{39}NO$	$C_{30}H_{29}NO$	C29H25N	C32H31ClCuN
For. weight	466,03	461.62	475.64	489.67	419.54	387,52	528,60
Crystal system	monoclinic	orthorhombic	triclinic	triclinic	monoclinic	monoclinic	triclinic
Space group	P2(1)/n	$P2_12_12_1$	P-1	P-1	$P2_1/c$	P 2_1/c	P -1
a(Å)	8.7777(5)	9.60582(12)	9.4189(2)	9.8121(3)	17.9140(9)	8.6700(7)	10.5711(15)
b(Å)	20.8566(11)	15.77345(14)	15.7194(5)	18.1535(7)	8.7789(4)	16.1199(12)	10.7188(15)
c(Å)	14.9695(10)	17.66478(17)	18.8016(5)	18.8637(7)	16.2084(8)	14.7922(11)	12.6875(18)
α(°)	90	90	89.976(2)	118.639(4)	90	90	91.360(4)°
β(°)	106.570(6)	90	89.574(2)	103.136(3)	111.964(6)	94.509(2)	96.550(4)°
γ(°)	90	90	86.602(2)	91.423(3)	90	90	116.012(4)°
V($Å^3$)	2626.7(3)	2676.51(5)	2778.78(13)	2836.4(2)	2364.0(2)	2060.95	1279.17(30)
Z	4	4	4	4	4	2	2
D_x(g cm^{-3})	1.178	1.15	1.14	1.15	1.18	1.249	1.372
F(000)	992	992	1024	1056	896	824	552
μ(mm^{-1})	0.165	0.518	0.512	0.068	0.070	0.072	0.980
Reflections:							
collected	6931	10468	26027	24232	15889	18048	19244
unique (R_{int})	4165	5115 (0.013)	9788	12197	4156	4371	5526
with I>2σ(I)	2931	4961	8314	9658	2738	3095	4429
R(F) [I>2σ(I)]	0.083	0.036	0.082	0.049	0.055	0.044	0.048
wR(F^2) [I>2σ(I)]	0.233	0.100	0.197	0.119	0.122	0.103	0.112
R(F) [all data]	0.118	0.037	0.089	0.066	0.094	0.070	0.064
wR(F^2) [all data]	0.258	0.101	0.200	0.132	0.136	0.114	0.118
Goodness of fit	1.03	1.03	1.04	1.01	1.00	1.03	1.08
max/min Δρ (e·$Å^{-3}$)	2.16/-0.92	0.15/-0.15	0.31/-0.22	0.94/-0.36	0.25/-0.17	0.22/-0.22	0.83/-0.75

4) References

[S1]: B. Rao, H. Tang, X. Zeng, L. Liu, M. Melaimi, G. Bertrand, Angew. Chemie Int. Ed. 2015, 54, 14915–14919.

[S2] Rigaku Oxford Diffraction (**2015**) CrysAlis PRO (Version 1.171.38.41).

[S3] G. Sheldrick, *Acta Crystallographica Section A Foundations and Advances* **2015**, *71*, 3-8.

[S4] G. Sheldrick, *Acta Crystallographica Section C Structural Chemistry* **2015**, *71*, 3-8.
